# Mechanical, Thermal, and Electrical Properties of Graphene-Epoxy Nanocomposites—A Review

**DOI:** 10.3390/polym8080281

**Published:** 2016-08-04

**Authors:** Rasheed Atif, Islam Shyha, Fawad Inam

**Affiliations:** Department of Mechanical and Construction Engineering, Faculty of Engineering and Environment, Northumbria University, Newcastle upon Tyne NE1 8ST, UK; aatif.rasheed@northumbria.ac.uk (R.A.); islam.shyha@northumbria.ac.uk (I.S.)

**Keywords:** mechanical properties, thermal properties, electrical properties, graphene, epoxy, nanocomposites

## Abstract

Monolithic epoxy, because of its brittleness, cannot prevent crack propagation and is vulnerable to fracture. However, it is well established that when reinforced—especially by nano-fillers, such as metallic oxides, clays, carbon nanotubes, and other carbonaceous materials—its ability to withstand crack propagation is propitiously improved. Among various nano-fillers, graphene has recently been employed as reinforcement in epoxy to enhance the fracture related properties of the produced epoxy–graphene nanocomposites. In this review, mechanical, thermal, and electrical properties of graphene reinforced epoxy nanocomposites will be correlated with the topographical features, morphology, weight fraction, dispersion state, and surface functionalization of graphene. The factors in which contrasting results were reported in the literature are highlighted, such as the influence of graphene on the mechanical properties of epoxy nanocomposites. Furthermore, the challenges to achieving the desired performance of polymer nanocomposites are also suggested throughout the article.

## 1. Introduction

Polymer Matrix Composites (PMCs) have found extensive applications in aerospace, automotive, and construction, owing to ease of processing and high strength-to-weight ratio, which is an important property required for aerospace applications [1]. Among different polymers, epoxy is the most commonly used thermosetting polymer matrix in PMCs [2]. The damage tolerance and fracture toughness of epoxy can be enhanced with the incorporation of (nano-) reinforcement, such as metallic oxides [3,4,5], clays [6,7,8], carbon nanotubes (CNTs) [9,10,11], and other carbonaceous materials [12,13,14]. After the groundbreaking experiments on the two-dimensional material graphene by Nobel Laureates Sir Andre Geim and Konstantin Novoselov [15] from the University of Manchester, graphene came into the limelight in the research community, mainly because of its excellent electrical [16], thermal [17], and mechanical properties [18]. Graphene found widespread applications in electronics [19], bio-electric sensors [20], energy technology [21], lithium batteries [22], aerospace [23], bio-engineering [24], and various other fields of nanotechnology [25]. There is an exponential rise in the use of graphene in different research areas, mainly because of the properties inherited in, and transferred by, graphene to the processed graphene-based materials.

To summarize the research trends related to graphene-based nanocomposites, multiple review articles were recently published in which various aspects of graphene-based nanocomposites were discussed. There are numerous ways to produce and characterize graphene-based materials [26]. Graphene-based materials were studied for different properties, such as thermal properties [27], mechanical properties [28], electrical properties [29], rheological properties [30], microwave adsorption [31,32], environmental and toxicological impacts [33], effect of preparation [34], and gas barrier properties [35]. These materials have found biological applications, especially related to toxicity [36], and in other applications like electrically-conductive adhesives [37] and selective photoredox reactions [38]. Because of their hierarchical pore structures, these materials were found suitable for gas sorption, storage, and separation [39]. Various factors influence the mechanical properties of graphene-based materials—e.g., γ-ray irradiation was found to have a strong influence on the structure–property relationship [40]. Various theoretical models were developed to predict the mechanical properties of epoxy–graphene nanocompsites and correlated with interphases and interfacial interactions [41]. It was presented that continuum mechanics can be used to predict the minimum graphene sheet dimensions and optimum number of layers for good reinforcement [42]. Graphene was compared with other reinforcements, such as clays [43] and CNTs [44], and was shown to have properties superior to the other nano-fillers. Various surface modifications were employed to improve interfacial interactions, and their influence on the performance of polymer nanocomposites was studied [45].

To date, eclectic reviews on graphene composites are covering a broad range of graphene-related issues; it can, however, be observed that there is an obvious gap in the lack of a review article discussing the mechanical, thermal, and electrical properties of epoxy–graphene nanocomposites. Therefore, this review article discusses the correlation between graphene structure, morphology, weight fraction, dispersion, surface modifications, and the corresponding mechanical, thermal, and electrical properties of epoxy–graphene nanocomposites.

## 2. Epoxy as Matrix

There are various types of epoxy which have a wide range of applications because of their superior attributes, such as improvement in composite mechanical properties, acceptable cost, and processing flexibility [2]. Phenolic glycidyl ethers are formed by the condensation reaction between epichlorohydrin and a phenol group. Within this class, the structure of the phenol-containing molecule and the number of phenol groups per molecule distinguish different types of resins and the final properties of monolithic epoxies and nanocomposites [2]. The epoxies have found some “high-end” applications, including aerospace, marine, automotive, high-performance sports equipment (such as tennis rackets), electronics, and industrial applications [46]. Due to the superior properties of carbonaceous materials, such as high strength and stiffness, they are most widely used at present as reinforcement in advanced Epoxy Matrix Composites (EMCs) [47,48,49,50].

Epoxy resins are of particular interest to structural engineers because these resins provide a unique balance of chemical and mechanical properties combined with extreme processing versatility [51]. When a composite is produced from epoxy-carbon using hand lay-up process, a great flexibility in aligning the fraction of fibers in a particular direction is available, which is dependent upon the in-service load on the composite structural member. In-plane isotropy can also be achieved by stacking the resin-impregnated fiber layers at equal numbers of 0°, +45°, −45°, and 90°. There are also other stacking sequences that can be used to achieve in-plane isotropy. The specific stiffness of quasi-isotropic epoxy–graphite laminated composite is higher than many structural metals. The highest specific strength achieved in epoxy–graphite is higher than common structural metals, with the exception of ultrahigh-strength steels and some β-titanium alloys. For example, the epoxy-carbon crutch is 50% lighter and still stronger than the aluminium crutch [2].

## 3. Graphene as Reinforcement

Graphene—a densely packed honey-comb crystal lattice made of carbon atoms having a thickness equal to the atomic size of one carbon atom—has revolutionized the scientific parlance due to its exceptional physical, electrical, and chemical properties. The graphene now found in various applications was previously considered only a research material and a theoretical model to describe the properties of other carbonaceous materials such as fullerenes, graphite, Single-Walled Carbon Nanotubes (SWNTs), and Multi-Walled Carbon Nanotubes (MWNTs). It was believed that the real existence of stand-alone single layer graphene would not be possible because of thermal fluctuations, as the stability of long-range crystalline order found in graphene was considered impossible at finite (room) temperatures. This perception was turned into belief by experiments when the stability of thin films was found to have direct relation with the film thickness; i.e., film stability decreases with a decrease in film thickness [52]. However, graphene can currently be found on a silicon substrate or suspended in a liquid and ready for processing. Although its industrial applications are not ubiquitous, it is widely used for research purposes (e.g., as reinforcement in PMCs) and has shown significant improvement in different (mechanical, thermal, electrical etc.) properties of produced nanocomposites [52,53,54,55,56].

The ability of a material to resist the propagation of an advancing crack is vital to the prevention of failure/fracture [57]. Graphene can significantly improve fracture toughness of epoxy at very low volume fraction by deflecting the advancing crack in the matrix. The details of the influence of various kinds of graphene/graphite nanoplatelets (GNPs) on the fracture toughness of epoxy nanocomposites are listed in Table 1. In all the composite systems mentioned in Table 1, epoxy was used as matrix and the nanocomposites were produced using solution casting technique, except [58] where the resin infiltration method was employed. The incorporation of graphene in epoxy can increase its fracture toughness by as much as 131% [59]. It can also be observed that graphene size, weight fraction, surface modification, and dispersion mode have strong influence on the improvement in fracture toughness values of the produced epoxy–graphene nanocomposites. Monolithic epoxy shows brittle fracture and beeline crack propagates, which results in straight fracture surfaces. The advancing crack in epoxy interacts with the graphene sheets. Initially, the crack propagates through the epoxy matrix as there are no significant intrinsic mechanisms available in monolithic epoxy to restrict crack propagation. However, no sooner than the crack faces strong graphene sheets ahead, it surrenders and subdues. Nevertheless, the extent of matrix strengthening and crack bridging provided by graphene strongly depends upon its dispersion state and interfacial interactions with the epoxy matrix [60,61].

## 4. Fracture Toughness

The successful employment of epoxy-based nanocomposites relies on the ability of the composite system to meet design and service requirements. The epoxy-based nanocomposites have found applications in aerospace, automotive, and construction due to ease of processing and high strength-to-weight ratio. In many applications, the composite system undergoes external loadings. The relationship between loads acting on a system and the response of the system towards the applied loads is studied in terms of mechanical properties. Therefore, epoxy-based nanocomposites are supposed to have superior mechanical properties. There are various tests to measure mechanical properties, such as tensile testing, bend testing, creep testing, fatigue testing, and hardness testing, to name a few. These tests usually take specimens of specific geometries and subject to loading at certain rate. In general, the industrial scale samples contain porosity and notches which act as stress concentrators and are deleterious to the mechanical properties of nanocomposites. Sometimes, it becomes difficult to control the maximum flaw size. The shape of the flaw is another very important parameter, as pointed notch (V-notch) is more detrimental than round notch (U-shaped) [62].

**Figure 1:**
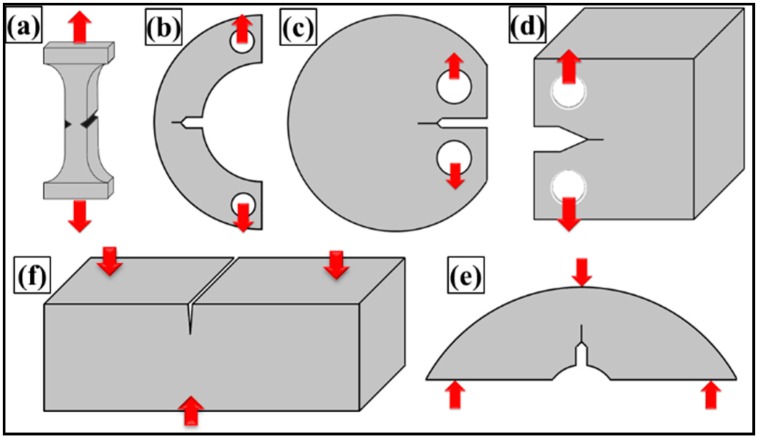
Various fracture toughness test specimen geometries: (**a**) notched tensile; (**b**–**d**) compact tension; (**e**) compact bend; and (**f**) single-edge notched three-point bend specimens. The arrows indicate the axis of loading.

**Figure 2:**
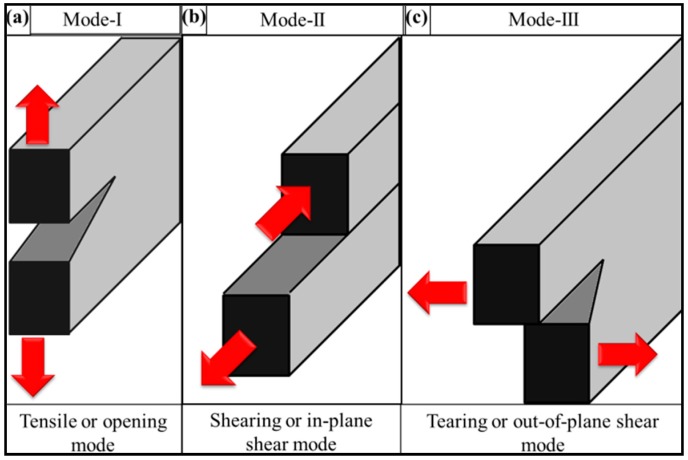
Various fracture modes: (**a**) mode-I, (**b**) mode-II, and (**c**) mode-III.

Due to the pronounced effect of defects on nanocomposite properties, it is important to understand how a system will tolerate external loading in the presence of a flaw under operating conditions, and how a system will resist the propagation of cracks from these flaws. Therefore, how the material will behave in reality will only be determined when the test specimen contains possible flaws, such as a notch. To deal with this issue in a pragmatic way, an intentional notch is produced in the specimen, and resistance to fracture is measured and is termed fracture toughness. Different specimens are used for fracture toughness, such as notched tension, three-point bending, and compact tension specimen, as shown in Figure 1. The toughness is usually measured in three different modes namely (1) Mode-I (tensile mode); (2) Mode-II (shearing mode); and (3) Mode-III (tearing mode), as shown in Figure 2. Most of the literature on epoxy nanocomposites reported Mode-I fracture toughness. Mode-I is preferred in contrast to Mode-II, because shear yielding is the dominant mechanism of failure that is acting under Mode-II, delivering higher values than in Mode-I. Mode-III is never practiced.

Some of the fracture toughness tests include double torsion, indentation, double cantilever tests, and Chevron notch method. Chevron notch method is popular, as it uses a relatively small amount of material and no material constants are needed for the calculations. The technique is also suitable for high-temperature testing, as flaw healing is not a concern. However, it requires a complex specimen shape that incurs an extra machining cost. The most commonly used specimen is a single-edge notched beam subjected to three or four-point bending. Unfortunately, it has been reported that the results of this test are very sensitive to the notch width and depth. Therefore, a pre-notched or molded beam is preferred. As polymers and polymer nanocomposites can be molded into a desired shape, a specific kind of notch can be replicated in multiple specimens. Due to the reproducibility of notch dimensions, the single-edge notched beam test can give reproducible values of fracture toughness in polymers and polymer nanocomposites. These are the reasons that most of the literature published on polymers and polymer nanocomposites used single-edge notch beams (subjected to three-point bend loading) to determine fracture toughness values (*K*_1C_). Impact loading methods, such as Charpy and Izod impact tests, are also used to determine impact fracture toughness. Fracture toughness values obtained through different techniques cannot be directly compared [91].

Fracture can be defined as the mechanical separation of a solid owing to the application of stress. Ductile and brittle are the two broad modes of fracture, and fracture toughness is related to the amount of energy required to create fracture surfaces. In ideally-brittle materials (such as glass), the energy required for fracture is simply the intrinsic surface energy of the materials, as demonstrated by Griffith [92]. For structural alloys at room temperature, considerably more energy is required for fracture, because plastic deformation accompanies the fracture process. In polymer nanocomposites, the fracture path becomes more tortuous as cracks detour around strong reinforcement. This increase in crack tortuosity provides additional work to fracture and, therefore, an increase in fracture toughness. In polymers, the fracture process is usually dominated by crazing or the nucleation of small cracks and their subsequent growth [93].

Toughness is defined as the ability of a material to absorb energy before fracture takes place. It is usually characterized by the area under a stress–strain curve for a smooth (un-notched) tension specimen loaded slowly to fracture. The term fracture toughness is usually associated with the fracture mechanics methods that deal with the effect of defects on the load-bearing capacity of structural components. The fracture toughness of materials is of great significance in engineering design because of the high probability of flaws being present. Defined another way, it is the critical stress intensity at which final fracture occurs. The plane strain fracture toughness (critical stress intensity factor, *K*_1C_) can be calculated for a single-edge notched three-point bending specimen using Equation (1), where *P*_max_ is the maximum load of the load–displacement curve (*N*), *f*(a/w) is a constant related to the geometry of the sample and is calculated using Equation (2), B is sample thickness (mm), *W* is sample width (mm), and *a* is crack length (*a* should be kept between 0.45 *W* and 0.55 *W*, according to ASTM D5045) [72]. The critical strain energy release rate (*G*_1C_) can be calculated using Equation (3), where E is the Young’s modulus obtained from the tensile tests (MPa), and *ν* is the Poisson’s ratio of the polymer. The geometric function *f(a*/*W*) strongly depends on the *a*/*W* ratio [94].

**Figure 3:**
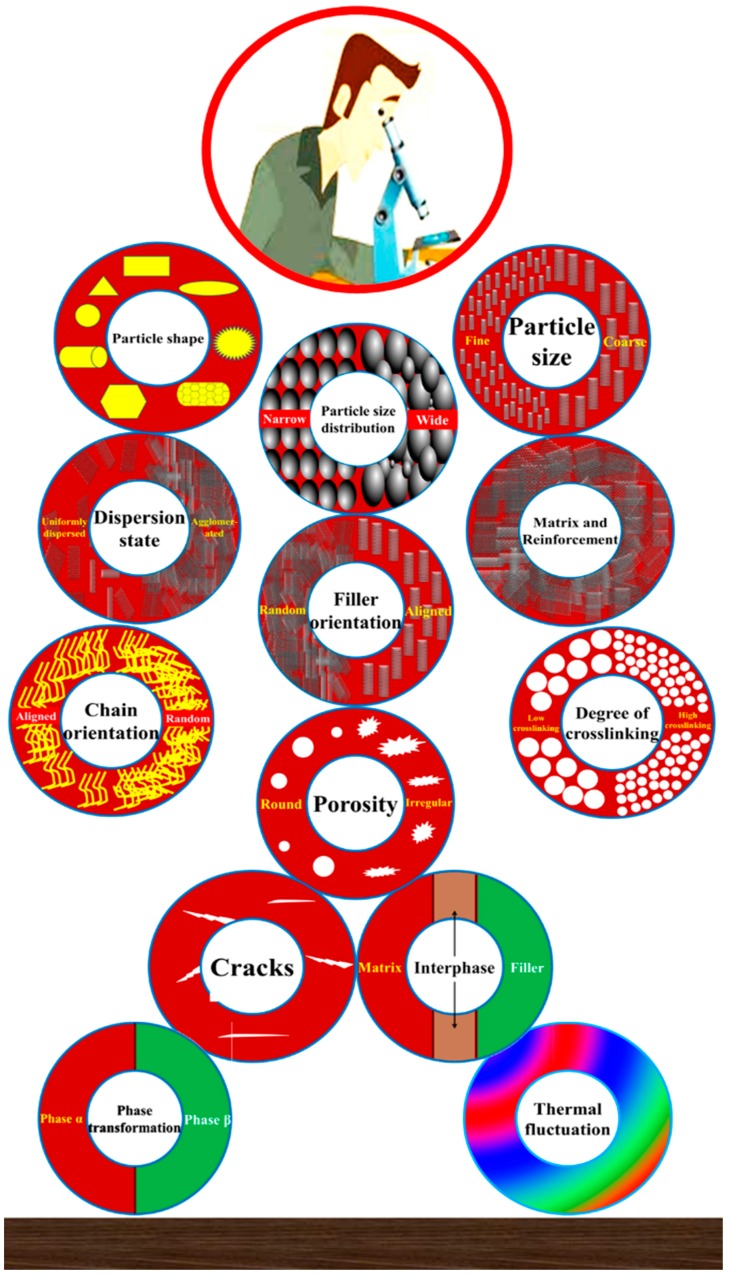
Various aspects of microstructure.

The fracture toughness is dependent on many factors, such as type of loading and environment in which the system will be loaded [95]. However, the key defining factor is the microstructure as summed up in Figure 3 [96]. The properties of nanocomposites are also significantly dependent on filler shape and size [51]. The graphene size, shape, and topography can be controlled simultaneously [97].

(1) $$K_{1C}=\frac{P_{\max}f\left(\frac{a}{W}\right)}{BW^{1/2}}$$

(2) f(aW)=[(2+aW){0.0866+4.64(aW)−13.32(aW)2+14.72(aW)3−5.6(a  W)4}](1−aW)3/2

(3) $$G_{1c}=\frac{K_{1c}^{2}\left(1-\nu^{2}\right)}{E}$$

## 5. Structure and Fracture Toughness

Graphene has a honeycomb lattice having sp^2^ bonding, which is much stronger than the sp^3^ bonding found in diamond [98]. There is sp^2^ orbital hybridization between *P*_x_ and *P*_y_ that forms a σ-bond [52]. The orbital *P*_z_ forms a π-bond with half-filled band that allows free motion of electrons. When bombarded with pure carbon atoms, hydrocarbons, or other carbon-containing molecules, the graphene directs the carbon atoms into vacant seats, thereby self-repairing the holes in the graphene sheet. Through their crack deflection modeling, Faber and Evans showed that maximum improvement in fracture toughness, among all other nano-reinforcements, can be obtained using graphene—mainly because of its better capability of deflecting the propagating cracks [99,100].

As graphene is a 2D structure, each carbon atom can undergo chemical reaction from the sides, resulting in high chemical reactivity. The carbon atoms on the edge of graphene sheet have three incomplete bonds (in single layer graphene) that impart especially high chemical reactivity to edge carbon atoms. In addition, defects within a graphene sheet are high energy sites and preferable sites for chemical reactants. All these factors make graphene a very highly chemical reactive entity. The graphene oxide can be reduced by using Al particles and potassium hydroxide [101]. The graphene structure can be studied using Transmission Electron Microscopy (TEM) and other high-resolution tools. Wrinkles were observed in graphene flat sheet, which were due to the instability of the 2D lattice structure [72,102].

Wrinkling is a large and out-of-plane deflection caused by compression (in-plane) or shear. Wrinkling is usually found in thin and flexible materials, such as cloth fabric [103]. Graphene nanosheets (GNSs) were also found to undergo a wrinkling phenomenon [104]. When wrinkling takes place, strain energy is stored within GNSs which is not sufficient to allow the GNSs to regain their shape. Wrinkling can be found on GNSs as well as on exfoliated graphite. The wrinkles in GNSs are sundering apart at different locations while getting closer at other regions. As GNSs do not store sufficient elastic strain energy, wrinkling is an irreversible phenomenon, but can be altered by external agency [105]. The surface roughness varies depending on graphene sheets, owing to their dissimilar topographical features, such as wrinkles’ size and shape. Therefore, the ability of sheets to mechanically interlock with other sheets and polymer chains is dissimilar. Wang et al. showed that a wrinkle’s wavelength and amplitude are directly proportional to sheet size (length, width, and thickness), as is clear from Equations (4) and (5), where λ is wrinkle wavelength, *ν* is Poisson’s ratio, *L* is graphene sheet size, *t* is thickness of graphene sheet, ε is edge contraction on a suspended graphene sheet, and A is wrinkle amplitude [57].

Palmeri et al. showed that the graphene sheets have a coiled structure that helps them to store a sufficient amount of energy [106]. The individual sheet and chunk of sheets together are subjected to plastic deformation at the application of external load. The applied energy is utilized in undertaking plastic work that enhances the material’s ability to absorb more energy. It is believed that large graphene sheets have large size wrinkles [107]. These wrinkles twist, bend, and fold the graphene sheets. The wrinkles and other induced defects remain intact while curing of polymer matrix. This reduces the geometric continuity and regularity of graphene and lowers load transfer efficiency, and can cause severe localized stress concentration.

(4) $$\lambda^{4}\approx \frac{4\pi^{2}\nu L^{2}t^{2}}{3\left(1-\nu^{2}\right)\varepsilon }$$

(5) $$A^{4}\approx \frac{16\nu L^{2}t^{2}\varepsilon }{3\pi^{2}\left(1-\nu^{2}\right)}$$

## 6. Surface Area and Fracture Toughness

*K*_1C_ strongly depends upon the surface area of the reinforcement, as it influences the matrix–reinforcement interfacial interactions. When the reinforcement has a large surface area, the interfacial area increases, which increases the number of routes for the transport of load from matrix to reinforcement [87]. On the contrary, when agglomeration takes place, not only the agglomerates act as stress raisers, but the net surface area is also decreased, which further drops the fracture toughness and other mechanical properties of nanocomposites [108]. One reason that graphene supersedes other reinforcements is its high surface area [109]. The surface area of graphene is even higher than that of CNTs [110]. To make a comparison, the surface areas of short carbon fiber and graphene are calculated. The surface area of carbon fiber is calculated using the formula for a solid cylinder, while the surface area of graphene is calculated using the formula for a rectangular sheet. The thickness of graphene is considered variable, so the same relation can be used for multiple layers of graphene sheets stacked together in the form of graphene nanosheets. The length of short carbon fiber is taken to be 1 µm and the diameter 0.1 µm. The dimensions of graphene are ℓ × *w* × *t* = 1 µm × 0.1 µm × 10 nm. The density of both short carbon fiber and graphene is taken to be 2.26 to make comparison based on dimensions only. The surface area of 1 g of carbon fibers is 19 m^2^ and that of graphene is 98 m^2^. It can be observed that although the lengths of both reinforcements are the same and the width of graphene is equal to the diameter of a short carbon fiber, there is a large difference in surface areas when the thickness of graphene is kept 10 nm. This difference will further increase if graphene dimensions are reduced. The specific surface area of graphene is as high as 2600 m^2^/g [111,112]. It shows that graphene, having a much larger surface area, can significantly improve the fracture toughness of the epoxy nanocomposites [113,114]. There is also improved thermal conduction among graphene–graphene links that significantly improves the overall thermal conductivity of the nanocomposites [115,116]. The electrical conductivity also increases with graphene as graphene sheets form links and provide a passageway for electrical conduction [117].

Zhao and Hoa used a theoretical computer simulation approach to study the improvement in toughness when epoxy is reinforced with 2D nano-reinforcements of different particle size [118,119]. The simulation results showed that there is a direct relation between particle size and stress concentration factor up to 1 µm, after which point the stress concentration factor was impervious to any further size increase. However, Chatterjee et al. [82] showed that fracture toughness was improved by increasing the graphene size, which is in negation with simulation results by Zhao and Hoa [120,121].

The relationship between graphene size and stress concentration factor can be correlated with the facile analogy of substitutional solid solution. The extent of strain field produced by a foreign atom depends upon the difference in atomic sizes of the foreign and parent atoms. When there is a large difference between foreign and parent atoms, a large strain field around the atom is generated. On the contrary, when the difference in atomic sizes of parent and foreign atoms is small, the strain field is limited. As both atomic and GNPs sizes are in the nano-meter range, the analogy can arguably be applied to an epoxy–graphene system where large sheet size will cause higher stress concentration factor than that produced by small sheet size. Therefore, graphene with smaller sheet size can be more efficient in improving the fracture toughness than the larger graphene sheets.

The increase in the fracture toughness of epoxy was found to be strongly dependent upon the graphene sheet size [57]. For the nanocomposites, an inverse relation was found between sheet size and fracture toughness in most cases. The increase in fracture toughness with a decrease in sheet size can be explained on the basis of stress concentration factor, as discussed above. Although graphene acts as reinforcement, however, it has associated stress and strain fields which arise from the distortion of the structure of polymer matrix. When sheet size, weight fraction, or both are increased beyond a certain value, the stress concentration factor dominates the reinforcing character. As a result, fracture toughness and other mechanical properties—such as tensile and flexural strength and stiffness—start decreasing, which is in accordance with Zhao and Hoa’s simulation results [118].

Wang et al. used Graphene Oxide (GO) of three different sizes, namely GO-1, GO-2, GO-3, having average diameters 10.79, 1.72, and 0.70 µm, respectively, to produce nanocomposites using an epoxy matrix [57]. They observed that fracture toughness was strongly dependent on GO sheet size. The maximum increase in fracture toughness was achieved with the smallest GO sheet size. The *K*_1C_ values dropped when weight fraction was increased beyond 0.1 wt %. This decrease in *K*_1C_ with increasing weight fraction can be correlated with crack generation and dispersion state.

## 7. Weight Fraction and Fracture Toughness

The *K*_1C_ first increases with GO and then starts decreasing in all three of the cases. The increase in *K*_1C_ is due to the reinforcing effect of GO, while the drop in *K*_1C_ is due to crack generation and agglomeration. The addition of a high GO weight fraction generates cracks that reduce the fracture toughness of the nanocomposite [57]. The other reason for such behavior is due to the high probability of agglomeration at higher weight fractions due to Van der Waals forces [57].

**Figure 4:**
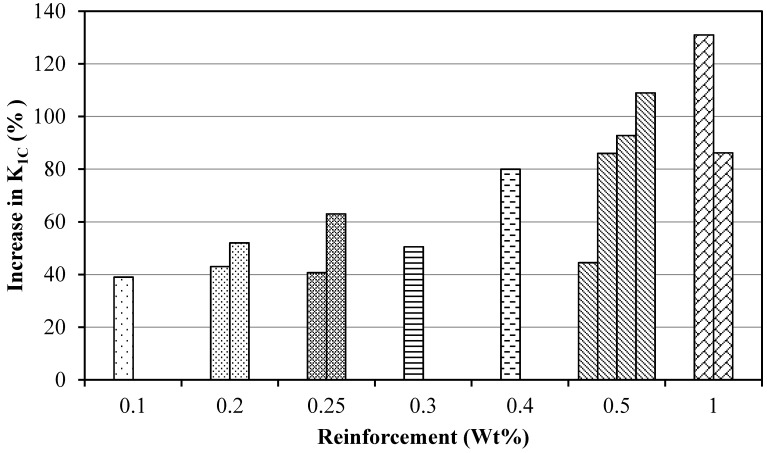
The weight fractions of reinforcements at which maximum *K*_1C_ was achieved in different epoxy/graphene nanocomposites and corresponding improvement (%) in *K*_1C_ (See references in Table 1).

The weight fractions of reinforcements at which maximum *K*_1C_ was achieved for different epoxy–graphene nanocomposites are shown in Figure 4. All the published research articles stated that the maximum *K*_1C_ values were achieved at or below 1 wt % of graphene, and *K*_1C_ dropped when the weight fraction of graphene was raised beyond 1 wt %. The decrease in *K*_1C_ with a higher weight fraction of graphene can be correlated with the dispersion state of graphene. As graphene weight fraction increases beyond 1 wt %, the dispersion state becomes inferior. The maximum increase in *K*_1C_ was 131%, which is achieved at 1 wt % graphene [59]. However, the dispersion mode adopted is worth discussing. The graphene was dispersed using a combination of sonication and mechanical stirring. This combination provides an efficient means of dispersing the graphene into epoxy. In addition to that, sonication causes exfoliation, delayering, and length shortening of graphene sheets. These aspects help alleviate the stress concentration factor and cracks associated with large graphene sheets. These factors result in *K*_1C_ improvement up to 131%, which is the maximum among the improvements in *K*_1C_ values reported in epoxy–graphene nanocomposites.

It can be observed from Figure 4 that there is no fixed value of GNPs wt % at which a maximum increase in K_1C_ is achieved. In addition, the increase in K_1C_ at fixed GNP wt % is not the same. For example, at 0.5 wt %, the % increase in *K*_1C_ is reported to be up to 45% by Chandrasekaran et al. [67], and about 110% by Ma et al. [80]. Therefore, it can be concluded that the wt % of GNPs is not the sole factor defining the influence of GNPs on the mechanical properties of nanocomposites. There are other influential factors as well, such as dispersion method, use of dispersant, and functionalization. In addition, the use of organic solvent is another important parameter in defining the improvement in mechanical properties. It was observed that a lower improvement in *K*_1C_ was observed when dispersion was carried out with only sonication, and a higher improvement in *K*_1C_ was observed when sonication was assisted with a secondary dispersion method, especially mechanical stirring.

## 8. Dispersion State and Fracture Toughness

**Figure 5:**
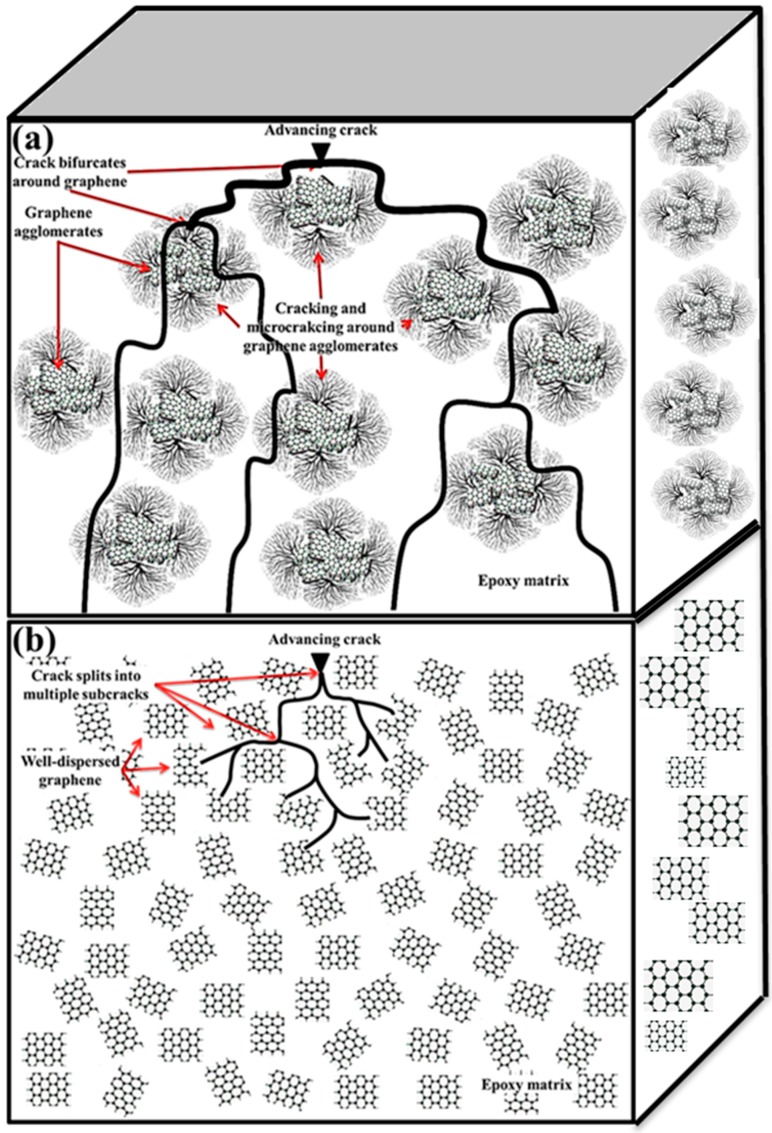
Influence of graphene dispersion on crack propagation method; (**a**) poorly dispersed graphene; (**b**) Ideally uniformly dispersed graphene. The arrows indicate the path followed by cracks through the graphene sheets.

The end product of most of the graphene synthesis methods is agglomerated graphene [33]. In addition, graphene tends to agglomerate due to weak intermolecular Van der Waals forces [113]. Therefore, dispersing graphene in epoxy matrix is always a challenge. The relationship between dispersion state and the nature of crack advancement is shown schematically in Figure 5. The advancing cracks can be best barricaded by uniformly dispersed graphene. Tang et al. produced highly dispersed and poorly dispersed RGO-epoxy nanocomposites using solution casting technique. The high dispersion of RGO in epoxy was achieved using a ball milling process [72]. The RGO dispersed in epoxy using sonication process and not subjected to ball milling was termed poorly dispersed. They studied the influence of graphene dispersion on the mechanical properties of the produced nanocomposite. The highly dispersed RGO-epoxy showed a 52% improvement in *K*_1C_, while the poorly dispersed RGO-epoxy showed only a 24% improvement in K_1C_. It shows that better dispersion of graphene can significantly improve the fracture toughness of epoxy nanocomposites [72].

**Figure 6:**
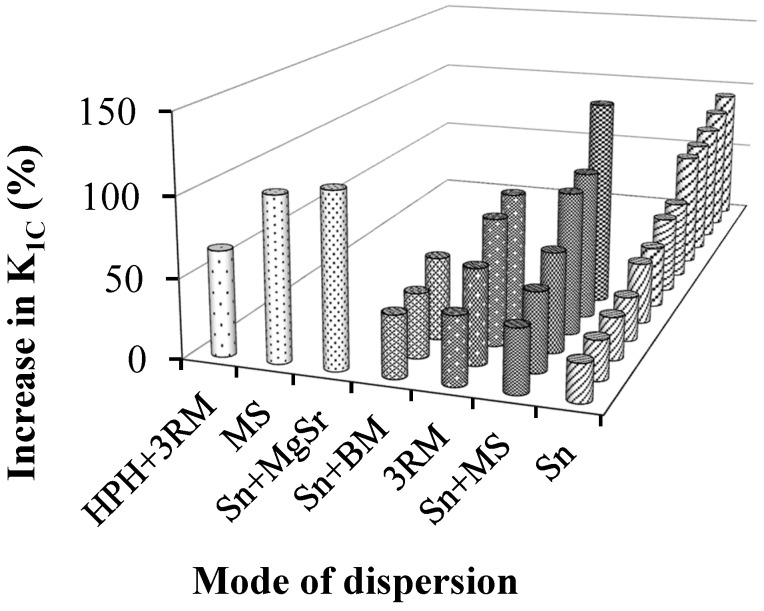
The maximum improvement in K_1C_ as a function of dispersion mode. (See references in Table 1).

Several dispersion modes to disperse reinforcement into epoxy matrix were successfully adopted (see references in Table 1). The maximum % increase in *K*_1C_ as a function of dispersion mode is shown in Figure 6. In most of these articles, sonication is the main mode of dispersing reinforcement in epoxy matrix. It can be observed that when sonication is assisted by a supplementary dispersion technique (such as mechanical stirring and magnetic stirring), the *K*_1C_ values were significantly increased. The maximum improvement of 131% in *K*_1C_ was achieved when a combination of sonication and mechanical stirring was employed [59]. The second highest improvement in *K*_1C_ was achieved with a combination of sonication and magnetic stirring, an increase in *K*_1C_ of 109% [80]. The minimum values in *K*_1C_ improvements are achieved when sonication is coupled with ball milling [60,64,100]. Since both the sonication and ball milling processes reduce the sheet size and produce surface defects [120,121,122,123,124,125,126,127,128,129,130,131,132,133,134], we believe that the surface defects significantly increased and sheet size was reduced below the threshold value, and therefore a greater improvement in *K*_1C_ was not achieved. Although three roll milling (3RM, calendering process) is an efficient way of dispersing the reinforcement into the polymer matrix due to high shear forces, the maximum improvement in K_1C_ using three roll mill was reported as 86% [77], which is far below that achieved with a combination of sonication and mechanical stirring (131% [59]).

## 9. Functionalization and Fracture Toughness

Achieving maximum improvement in fracture toughness of polymers by using graphene depends on the ability to optimize the dispersibility of graphene and the interfacial interactions with the epoxy matrix. As described previously, graphene tends to agglomerate due to the weak Van der Waals interactions, and its smoother surface texture inhibits strong interfacial interactions. To tackle the limited dispersibility and interfacial bonding of graphene, surface modifications are carried out [135,136,137,138,139]. In fact, the introduction of functional groups on the graphene surface can induce novel properties [140,141,142,143,144]. Various methods to modify the graphene surface have been employed, and can be categorized into two main groups, namely: (1) chemical functionalization; and (2) physical functionalization.

In chemical functionalization, chemical entities are typically attached covalently. For example, in defect functionalization, functional groups are attached at the defect sites of graphene, such as –COOH (carboxylic acid) and –OH (hydroxyl) groups. Defects can be any departure from regularity, including pentagons and heptagons in the hexagonal structure of graphene. Defects may also be produced by reaction with strong acids such as HNO_3_, H_2_SO_4_, or their mixture, or strong oxidants including KMnO_4_, ozone, and reactive plasma [145]. The functional groups attached at the defect sites of graphene can undergo further chemical reactions, including but not limited to silanation, thiolation, and esterification [146]. Unlike chemical functionalization, physical functionalization has non-covalent functionalization, where the supermolecular complexes of graphene are formed as a result of the wrapping of graphene by surrounding polymers [33]. Surfactants lower the surface tension of graphene, thereby diminishing the driving force for the formation of aggregates. The graphene dispersion can be enhanced by non-ionic surfactants in case of water-soluble polymers [33].

**Figure 7:**
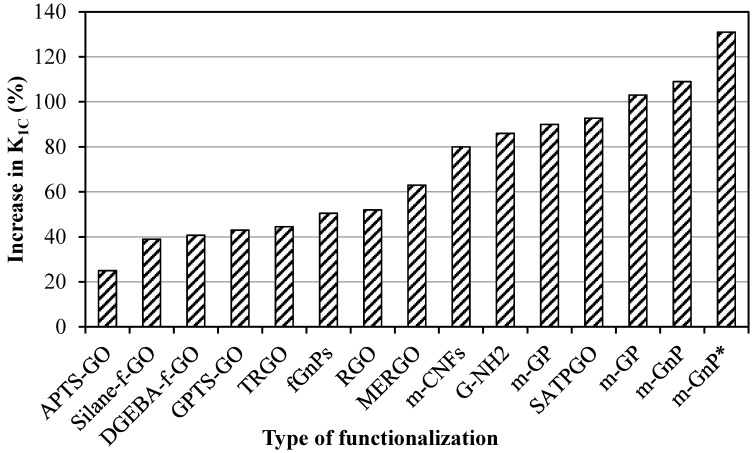
The maximum improvement in *K*_1C_ as a function of functionalization method. (See references in Table 1).

The different functionalization methods adopted to study their influence on *K*_1C_ values with corresponding improvements (%) in *K*_1C_ values are shown in Figure 7. The minimum improvement was achieved for amino-functionalized graphene oxide (APTS-GO) [74], while the maximum improvement was recorded for surfactant-modified graphene nanoplatelets [59]. This could be attributed to the improvement in the dispersion state of graphene in the epoxy matrix when surfactants were used, in addition to improving interactions without causing a reduction in graphene sheet size or imparting surface defects on graphene sheets.

## 10. Crosslink Density and Fracture Toughness

In thermosetting materials, such as epoxy, high crosslink density is desirable for the improvement of mechanical properties. However, high crosslink density has a detrimental effect on fracture toughness [57]. Therefore, a crosslink density threshold is required in order to achieve optimal properties [147,148]. During the curing of thermoset polymers, while phase transformation takes place, graphene sheets tend to agglomerate in order to reduce configurational entropy [57]. Additionally, the viscosity initially reduces when the temperature is increased during curing, which makes the movement of the graphene sheets relatively easy, supporting their agglomeration. Due to the wrinkle-like structure and high specific surface area of graphene, strong interfacial interactions are possible with epoxy chains. It may also affect the overall curing reaction by changing the maximum exothermic heat flow. Molecular dynamics studies conducted by Smith et al. also showed that there was a change in polymer chain mobility caused by geometric constraints at the surface of nano-reinforcement [149].

The graphene affects the crosslink density of epoxy [65]. When graphene is dispersed in epoxy, the polymer chains are restricted, and crosslinking is decreased. The decrease in crosslinking lowers the heat release rate. It was reported that both graphene platelets (GnPs) and polybenzimidazole functionalized GnPs (fGnPs) decreased the heat release rate of the curing reaction and increased the curing temperature [65]. It can also be attributed to the dispersion state of the reinforcement. Uniformly dispersed reinforcement will have a more pronounced effect on heat release rate and curing temperature than poorly dispersed reinforcement. Therefore, fGnPs have a better dispersion state than GnPs [65]. There are two opposite effects of filler in the matrix: (1) the fillers could restrict the polymer chains, which should increase *T*_g_; (2) the reactive fillers could lower the crosslinking density of epoxy, which should decrease *T*_g_. An increase in *T*_g_ shows that interfacial interactions dominate the crosslinking density effect [65].

## 11. Fracture Patterns

Monolithic epoxy exhibits a bamboo-like brittle fracture pattern [105]. However, with the incorporation of graphene, the cracks are deflected, resulting in parabolic and non-linear fracture patterns [105]. The change in graphene structure and shape upon the application of external stress also affects the overall fracture pattern of the nanocomposite, due to changes in mechanical interlocking and interfacial interactions [105]. It was recorded that bending behavior of GNSs when wrapping around a corner resulted in the sliding of layers over one another, and was termed “sliding mode” [105]. In sliding mode, angular change (γ) was observed. This γ was produced when layers slid over one another. If the state of stress is relatively high, the inner layers undergo splitting and buckling that further results in kinking, by which the bending stress is alleviated [105]. GNSs size and edge morphology control the type of fracture mode. In the case of smaller GNSs (smaller refers to volume of individual GNSs), where the sliding surface is smaller, the resistance to sliding is lower, and hence sliding mode will be preferred. On the contrary, if GNSs are of larger size and the sides are longer, the resistance to sliding would be higher, and hence buckling mode will be preferred over sliding mode [105]. The tearing step subdivides into multiple steps. Consequently, the initial crack branches into multiple small cracks [105]. However, the extent of subdivision of the advancing cracks depends on the dispersion state of the filler and interfacial interactions.

## 12. Other Mechanical Properties

**Figure 8:**
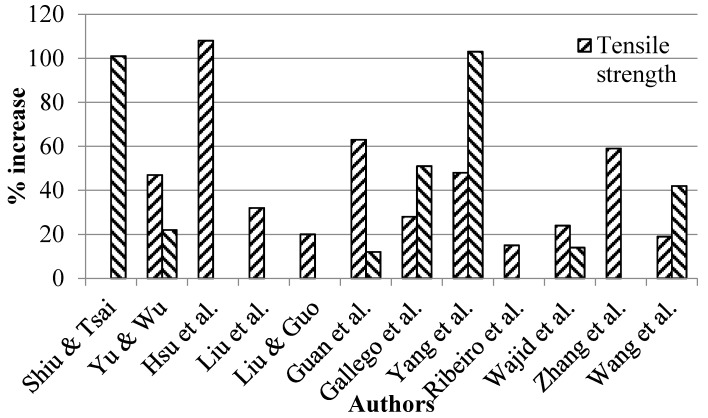
The % increase in tensile properties of epoxy/graphene nanocomposites [164,165,182,183,184,185,186,187,188,189,190,191].

The literature shows an absence of consensus on the role of graphene in improving other mechanical properties of nanocomposites. Some authors reported significant improvement in the mechanical properties of nanocomposites reinforced with GNPs [150,151,152,153,154]. On the other hand, there was no significant effect due to the incorporation of GNPs into epoxy matrix [155,156,157,158], and even worse, the mechanical properties deteriorated by the addition of GNPs [159,160,161,162,163]. In general, a major portion of the literature has shown that GNPs can significantly improve the mechanical properties of epoxy nanocomposites. The percent improvements in tensile strength and tensile modulus are shown in Figure 8. The maximum improvement in tensile strength is as high as 108% [164] and in the tensile modulus up to 103% [165]. GNPs were also found to improve flexural properties of nanocomposites. Naebe et al. produced covalent functionalized epoxy–graphene nanocomposites, and reported 18% and 23% increase in flexural strength and modulus, respectively [166]. Qi et al. produced graphene oxide–epoxy nanocomposites and reported an increase of up to 53% in flexural strength [167]. The impact strength and hardness were also significantly improved by graphene in epoxy nanocomposites. For example, Ren et al. applied a combination of bath sonication, mechanical mixing, and shear mixing to disperse GO in cyanate ester–epoxy and produced nanocomposites using in situ polymerization [168]. They reported an increase of 31% in impact strength. Qi et al. produced graphene oxide–epoxy nanocomposites and reported an increase in impact strength of up to 96% [169], whereas Lu et al. produced GO–epoxy nanocomposites and reported an increase in impact strength of up to 100% [170]. Shen et al. produced GNS–epoxy nanocomposites and reported an increase in impact strength of up to 11% [171], and Bao et al. reported an increase in hardness of up to 35% [172]. The G_1C_ also improved with the incorporation of graphene in epoxy nanocomposites. Meng et al. produced epoxy–graphene nanocomposites and reported an increase in G_1C_ of up to 597% [173].

## 13. Thermal Properties

Due to the superior thermal conductivity of graphene, graphene-based polymer nanocomposites are promising candidates for high-performance thermal interface materials [174]. The dissipation of heat from electronic devices may also be barricaded when the high thermal conductivity of graphene is efficiently utilized. The graphene has shown higher efficiency in increasing the thermal conductivity of polymers than CNTs [175]. It has been found experimentally that the Effective Thermal Conductivity (*K*_eff_) of graphene-based polymer nanocomposites has a non-linear dependence on graphene weight fraction [176,177,178]. Xie et al. proposed an analytical model to determine the *K*_eff_ of graphene-based nanocomposites [179]. Their model proposed very high thermal conductivity values, as the model did not take into account the interfacial thermal resistance. Lin et al. developed a model based on Maxwell–Garnett effective medium approximation theory to determine the effective thermal conductivity of graphene-based nanocomposites [180,181]. They showed that the enhancement in thermal conductivity is strongly influenced by the aspect ratio and orientation of graphene.

**Figure 9:**
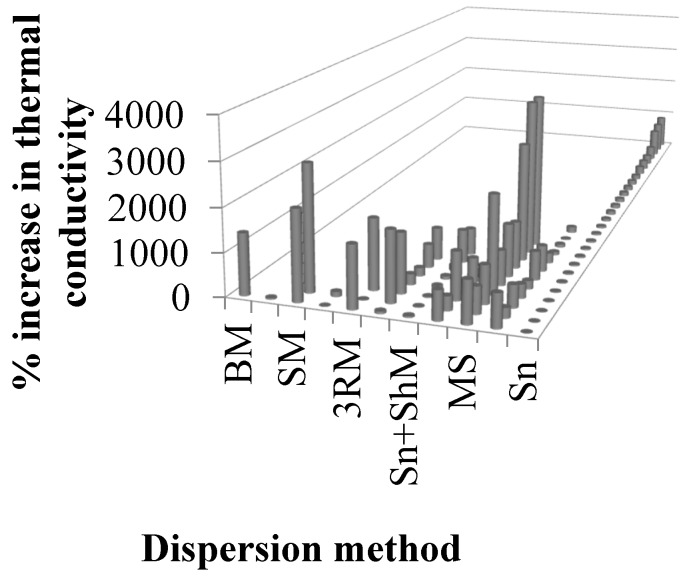
Percent increase in thermal conductivity as a function of dispersion method (see references in Table 2).

Hu et al. used a molecular dynamics approach to show that the agglomeration of graphene is of major concern in increasing the thermal conductivity of the system [192]. The variation in thermal conductivity with various forms of graphene and graphite nanocomposites is summarized in Table 2, and the influence of dispersion mode on the improvement of thermal conductivity is shown in Figure 9. The maximum improvement in thermal conductivity was observed in the case of mechanical stirring. In general, sonication caused a lower improvement in thermal conductivity. However, maximum improvement in thermal conductivity (not shown in Figure 9) was observed in the case of sonication, 1.6 × 10^4^% [193].

## 14. Electrical Properties

**Figure 10:**
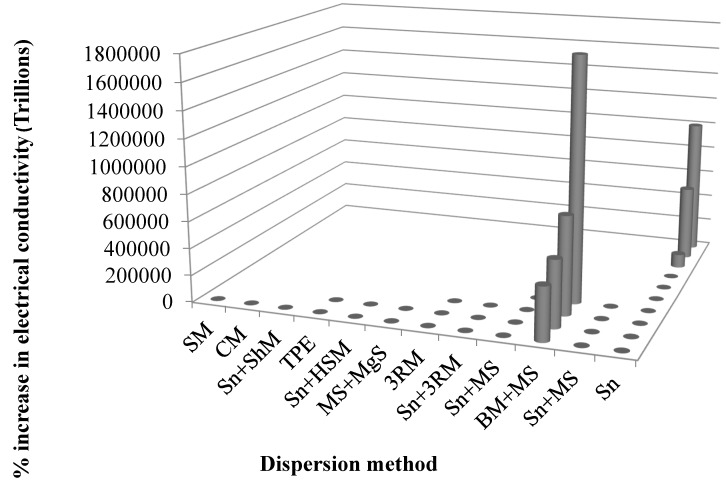
Percent increase in electrical conductivity as a function of dispersion method (see reference in Table 3).

Tailoring the electrical properties of graphene can unlock its many potential electronic applications [194,195]. For example, effective gauge fields are introduced when graphene lattice deformation takes place. Like the effective magnetic field, the produced effective gauge fields influence the Dirac fermions [196]. The Fermi level in undoped graphene lies at the Dirac point, where the minimum conductivity values are achieved [197]. By adding free charge carriers (i.e., dopants), the electrical properties of graphene can be improved, and conductivity increases linearly with carrier density [198,199]. For example, boron as dopant can contribute ~0.5 carriers per dopant in a graphene sheet [200]. Dopants can be introduced during the synthesis of graphene using chemical vapor deposition (CVD) [201]. The variation in electrical conductivity with various forms of graphene and graphite nanocomposites is summarized in Table 3, and the influence of dispersion mode on the improvement of thermal conductivity is shown in Figure 10. The maximum improvement in electrical conductivity was observed in the case of a combination of ball milling and mechanical stirring. Therefore, both thermal and electrical conductivities improved in the case of mechanical stirring.

## 15. Conclusions

The following are the key points related to epoxy/graphene nanocomposites:

1. Epoxy is an excellent matrix for graphene composites because of its efficient properties such as enhancement in composite mechanical properties, processing flexibility, and acceptable cost [2].
2. Graphene can significantly enhance the fracture toughness of epoxy nanocomposites—i.e., up to 131% [59]. When epoxy is reinforced with graphene, the carbonaceous sheets shackle the crack and restrict its advancement. This obstruction and deflection of the crack by the graphene at the interface is the foremost mechanism of raising the fracture toughness of nanocomposites.
3. The graphene sheets with smaller length, width, and thickness are more efficient in improving the fracture toughness than those with larger dimensions [57]. Large graphene sheets have a high stress concentration factor, because of which crack generation becomes easy in the epoxy matrix [118,119]. The cracks deteriorate the efficiency of graphene in enhancing the fracture toughness of epoxy/graphene nanocomposites.
4. Uniformly dispersed graphene improves fracture toughness significantly as compared to the poorly dispersed graphene [72]. It is evident from the published literature that the fracture toughness dropped when graphene weight fraction was increased beyond 1 wt %. The decrease in fracture toughness with higher weight fraction of graphene can be correlated with the dispersion state of graphene. As graphene weight fraction increases beyond 1 wt %, the dispersion state becomes inferior.
5. Three roll milling or calendering process is an efficient way of dispersing the reinforcement into a polymer matrix, as it involves high shear forces [244,245,246,247,248]. However, the maximum enhancement in fracture toughness was achieved with a combination of sonication and mechanical stirring [59].
6. In thermosetting materials such as epoxy, high crosslink density is desirable for improved mechanical properties. However, fracture toughness is dropped with high crosslinking [57].
7. The literature has proved the absence of consensus of graphene’s role in improving the mechanical properties of nanocomposites [150,151,152,153,154]. Generally, graphene acts as panacea and raises the mechanical properties [116,155,156,157,158]. On the contrary, it acts as placebo and shows no effect on mechanical properties. Even worse, it is inimical and razes the mechanical properties [160,161,162,163,164]. The main factors that dictate graphene’s influence on the mechanical properties of epoxy nanocomposites include topographical features, morphology, weight fraction, dispersion state, surface modifications, and interfacial interactions.

## Figures and Tables

**Table 1:** A brief record of epoxy-based nanocomposites studied for improvement in fracture toughness values.

Sr.	Authors	Year	Reinforcement/(wt %)		Dispersion method	% Increase in *K*_1C_ (MPa·m^1/2^)	Remarks	Ref.
1	Wan et al.	2014	GO (0.25 wt %)		Sn + BM	25.6	*K*_1C_ drops after 0.25 wt % of reinforcement	[63]
			DGEBA-f-GO (0.25 wt %)			40.7		
2	Sharmila et al.	2014	MERGO (0.25 wt %)		MS + USn	63	*K*_1C_ drops after 0.25 wt % of reinforcement	[64]
3	Zhang et al.	2014	GnPs (0.5 wt %)		Sn	27.6	Trend still increasing	[65]
			fGnPs (0.3 wt %)			50.5	*K*_1C_ drops after 0.3 wt % of reinforcement	
4	Moghadam et al.	2014	UG (0.5 wt %)		3RM	55	*K*_1C_ drops after 0.5 wt % of reinforcement	[66]
			GO (0.5 wt %)			57		
			G-NH_2_ (0.5 wt %)			86		
			G-Si (0.5 wt %)			86		
5	Ma et al.	2014	m-GnP (1 wt %)		MS + Sn	131	*K*_1C_ drops after 1 wt % of reinforcement of m-GnP	[59]
6	Chandrasekaran et al.	2014	TRGO (0.5 wt %)		3RM	44.5	Trend still increasing	[67]
			GNP (1 wt %)			49	*K*_1C_ drops after 1 wt %	
			MWCNTs (0.5 wt %)			12.7	Trend still increasing	
7	Wan et al.	2014	GO (0.1 wt %)		Sn + BM	24	*K*_1C_ improves with silane functionalization	[68]
			Silane-f-GO (0.1 wt %)			39		
8	Zaman et al.	2014	m-clay (2.5 wt %)		MS	38	*K*_1C_ drops after 2.5 wt % m-clay	[69]
			m-GP (4 wt %)			103	Trend still increasing	
9	Jiang et al.	2014	SATPGO (0.5 wt %)		USn	92.8	*K*_1C_ drops after 0.5 wt % of reinforcement	[70]
10	Shokrieh et al.	2014	GPLs (0.5 wt %)		Sn	39	*K*_1C_ drops after 0.5 wt % of reinforcement	[71]
			GNSs (0.5 wt %)			16		
11	Jia et al.	2014	GF (0.1 wt %) (resin infiltration)		None	70	*K*_1C_ did not change much between 0.1 to 0.5 wt %	[58]
12	Tang et al.	2013	Poorly dispersed RGO (0.2 wt %)		Sn	24	Trend still increasing	[72]
			Highly dispersed RGO (0.2 wt %)		Sn + BM	52		
13	Wang et al.	2013	GO	10.79 µm (0.5wt %)	USn	12	*K*_1C_ drops after 0.5 wt % of reinforcement	[57]
				1.72 µm (0.5 wt %)		61		
				0.70 µm (0.1 wt %)		75		
14	Chandrasekaran et al.	2013	GNPs* (0.5 wt %)		3RM	43	Dispersion and *K*_1C_ improved with three roll milling	[73]
15	Li et al.	2013	APTS-GO (0.5 wt %)		USn	25	Trend still increasing	[74]
			GPTS-GO (0.2 wt %)			43	*K*_1C_ drops after 0.2 wt % of reinforcement	
16	Shadlou et al.	2013	ND (0.5 wt %)		USn	No effect	Fracture toughness improvement is higher by CNF and GO (high aspect ratio) compared with that by spherical ND	[75]
			CNF (0.5 wt %)			4.3		
			GO (0.5 wt %)			39.1		
17	Jiang et al.	2013	GO (0.1 wt %)		Sn	31	Trend remains same after 1 wt % of reinforcement	[76]
			ATS (1 wt %)			58.6	*K*_1C_ drops after 0.1 wt % of reinforcement	
			ATGO (1 wt %)			86.2	The maximum improvement is achieved with functionalization	
18	Liu et al.	2013	p-CNFs (0.4 wt %)		Sn	41	Trend still increasing	[77]
			m-CNFs (0.4 wt %)			80		
19	Wang et al.	2013	ATP (1 wt %)		Sn	14	*K*_1C_ drops after 0.1 wt %	[78]
			GO (0.2 wt %)			19	Trend still increasing after 0.2 wt %	
			ATP (1 wt %) + GO (0.2 wt %)			27	*K*_1C_ drops with the further increase in ATP of reinforcement	
20	Alishahi et al.	2013	ND (0.5 wt %)		Sn	−26.9	Trend still increasing	[79]
			CNF (0.5 wt %)			19		
			GO (0.5 wt %)			23		
			CNT (0.5 wt %)			23.8		
21	Ma et al.	2013	U-GnP (0.5 wt %)		MgSr + USn	49	Trend still increasing	[80]
			m-GnP (0.5 wt %)			109		
22	Feng et al.	2013	Graphene (0.5 wt %)		Sn	76	*K*_1C_ decreases after 0.5 wt % of reinforcement	[81]
23	Chatterjee et al.	2012	GnPs (5 µm, 2 wt %)		3RM	60	Trend still increasing	[82]
			GnPs (25 µm, 2 wt %)			80		
			CNTs (2 wt %)			80		
			CNT:GnP = (9:1) (2 wt %)			76		
24	Chatterjee et al.	2012	EGNPs (0.1 wt %)		HPH + 3RM	66	*K*_1C_ drops after 0.1 wt % of reinforcement	[83]
25	Zaman et al.	2011	GP (2.5 wt %)		Sn + MS	57	The surface modification significantly improved the *K*_1C_	[84]
			m-GP (4 wt %)			90		
26	Rana et al.	2011	CNFs		Sn + MS	40	*K*_1C_ is dependent upon mixing time	[85]
27	Bortz et al.	2011	GO (0.5 wt %)		3RM	60	*K*_1C_ drops after 0.5 wt % of reinforcement	[86]
28	Zhang et al.	2010	CNFs (0.5 wt %)		3RM	19.4	Trend still increasing	[87]
			SCFs (15 wt %)			125.8		
			SCF (10 wt %)/CNF (0.75 wt %)			210		
29	Fang et al.	2010	GNs		MS + Sn	93.8	Better results with combination of MS and Sn	[88]
30	Jana et al.	2009	GP with “puffed” structure (5 wt %)		Sn	28	Trend still increasing	[89]
31	Rafiee et al.	2009	SWNT (0.1 wt %)		Sn + MS	17	Graphene platelets have more influence on *K*_1C_ than CNTs	[90]
			MWNT (0.1 wt %)			20		

3RM: three roll milling; APTS-GO: amino-functionalized graphene oxide (GO); ATGO: 3-Aminopropyltriethoxysilane functionalized silica nanoparticles attached GO; ATP: attapulgite; ATS: 3-amino functionalized silica nanoparticles; BM: ball milling; CNF: carbon nanofiber; CNT: carbon nanotube; DGEBA-f-GO: diglycidyl ether of bisphenol-A functionalized GO; EGNP: amine functionalized expanded graphene nanoplatelets; fGnP: polybenzimidazole functionalized graphene platelets (GnPs); G-NH2: amino-functionalized GNPs; G-Si: silane modified GNPs; GF: graphene foam; GN: amine functionalized graphene sheet; GnP: graphene platelet; GNP*: graphite nanoplatelet; GNS: graphene nanosheet; GO: graphite; GP: graphite particles; GPL: graphene nanoplatelets; GPTS-GO: epoxy functionalized GO; HPH: high pressure homogenizer; m-clay: surface modified nano clay; m-CNF: triazole functionalized carbon nanofiber; m-GnP: surface modified GnP; m-GnP*: surfactant modified graphene platelet; m-GP: surface modified graphene platelets; MERGO: microwave exfoliated reduced graphene oxide; MgSr: magnetic stirring; MS: mechanical stirring; MWCNT: multi-walled carbon nanotube; MWNT: multi-walled carbon nanotubes; ND: nanodiamond; pCNF: pristine carbon nanofibers; RGO: thermally reduced graphene oxide; SATPGO: 3-aminopropyltriethoxysilane modified silica nanoparticles attached GO; SCF: short carbon fibers; Silane-f-GO: silane functionalized GO; Sn: Sonication; SWNT: single-walled carbon nanotubes; U-GnP: unmodified graphene platelets; UG: unmodified graphene nanoplatelets; USn: ultrasonication.

**Table 2:** A brief record of epoxy-based nanocomposites studied for improvement in thermal conductivity values.

Sr.	Authors	Year	Reinforcement (wt %)	Dispersion method	% Increase in thermal conductivity	Remarks	Ref.
1	Kandre et al.	2015	GnP (1.9 wt %)	Sn	9	The simultaneous inclusion of GnPs and SnP/SnW at a combined loading of 1 vol % resulted in about 40% enhancement in the through-thickness thermal conductivity, while the inclusion of GnP at the same loading resulted in only 9% improvement. A higher increment with simultaneous addition of GnP and SnP/SnW can be attributed to synergistic effects.	[202]
			SnP/(0.09 wt %)		18		
			SnW/(0.09 wt %)		8		
			GnP (1.9 wt %), SnP (0.09 wt %)		38		
			GnP (1.9 wt %), SnW (0.09 wt %)		40		
2	Tang et al.	2015	Three-dimensional graphene network (3DGNs) (30 wt %)	None	1,900	(Composites produced using layer-by-layer dropping method.) The filler with large size is more effective in increasing the thermal conductivity of epoxy because of continuous transmission of acoustic phonons and minimum scattering at the interface due to reduced interfacial area. High intrinsic thermal conductivity of graphene is the major reason for the obtained high thermal conductivity of nanocomposites.	[203]
			Chemically reduced graphene oxide (RGO) (30 wt %)	Sn + MS	1,650		
			Natural graphite powder (NG) (30 wt %)		1,400		
3	Burger et al.	2015	Graphite flakes (12 wt %) (GRA-12)	Sn + MgSr	237.5	As the filler/matrix interfaces increase, the thermal resistance increases due to phonon scattering. In order to improve the thermal conductivity of a composite, it is better to structure a sample with an adapted morphology than trying to have the best dispersion. A 3D-network was first prepared with graphite foils oriented through the thickness of the sample and then stabilized with DGEBA/DDS resin. The produced composite sample was called as “Network”. In “fibers”, all the graphite flakes were aligned through the thickness of sample. When a DGEBA interface layer was applied in “fiber”, the sample was called “Fiber + 1 interface”. When two DGEBA interface layers was applied in “fiber” the sample was called as “Fiber + 2 interfaces”.	[204]
			Graphite flakes (15 wt %) (GRA-15)		325		
			Graphite flakes (14–15 wt %) (Network)		775		
			Graphite flakes (11–12 wt %) (Fibers)		666.7		
			Graphite flakes (11–12 wt %) (Fiber + 1 interface)		608.3		
			Graphite flakes (11–12 wt %) (Fiber + 2 interface)		237.5		
4	Zeng et al.	2015	Liquid crystal perylene bisimides polyurethane (LCPU) modified reduced graphene oxide (RGO) (1 wt %)	Sn	44.4	Along with the increase in thermal conductivity, the impact and flexural strengths increased up to 68.8% and 48.5%, respectively, at 0.7 wt % LCPU/RGO.	[205]
5	Wang et al.	2015	GnPs, 1 µm, (GnP-C750)	Sn + MgSr + 3RM	9.1	The increase in thermal conductivity is higher in the case of larger particle size than smaller particle size.	[206]
			GnPs, 5 µm		115		
6	Zhou et al.	2015	Multi-layer graphene oxide (MGO) (2 wt %)	Sn	95.5	The thermal conductivity decreases after 2 wt % MGO.	[207]
7	Zeng et al.	2015	Al_2_O_3_ nanoparticles (30 wt %)	Sn	50	The thermal conductivity can be improved by using hybrid fillers.	[208]
			Aminopropyltriethoxy-silane modified Al_2_O_3_ nanoparticles (Al_2_O_3_-APS) (30 wt %)		68.8		
			Liquid-crystal perylene-bisimide polyurethane (LCPBI) functionalized reduced graphene oxide (RGO) and Al_2_O_3_-APS (LCPBI/RGO/Al_2_O_3_-APS)		106.2		
8	Tang et al.	2015	Al_2_O_3_ (18.4 wt %)	Sn + MS	59.1	The increase in thermal conductivity decreases with Al_2_O_3_ coating of graphite.	[209]
			Graphite (18.4 wt %)		254.6		
			Al_2_O_3_-coated graphite (Al_2_O_3_-graphite) (18.4 wt %)		195.5		
9	Pan et al.	2015	Perylene bisimide (PBI)-hyper-branched polyglycerol (HPG) modified reduced graphene oxide (RGO), (PBI-HPG/RGO) (1 wt %)	Sn	37.5	The filler was observed to be uniformly dispersed, resulting in strong interfacial thermal resistance.	[210]
10	Wang et al.	2015	SiO_2_, 15 nm, (1 wt %)	Sn	14.3	SiO_2_ nanoparticles are more effective in increasing thermal conductivity than GO. The maximum improvement in thermal conductivity was observed in the case of hybrid filler.	[211]
			GO (1 wt %)		4.8		
			As-prepared nanosilica/graphene oxide hybrid (m-SGO) (1 wt %)		28.6		
11	Zha et al.	2015	GNPs (3.7 wt %), Al_2_O_3_ nanoparticles (ANPs), (65 wt %)	Sn + MS	550.4	Al_2_O_3_ nanofibers are more effective in improving thermal conductivity than Al_2_O_3_ nanoparticles.	[212]
			GNPs (3.7 wt %), Al_2_O_3_ fibers (Afs) (65 wt %)		756.7		
12	Zhou et al.	2015	Multi-layer graphene oxide (MGO) (2 wt %)	Sn	104.8	The thermal conductivity decreases after 2 wt % MGO.	[213]
13	Wang et al.	2015	GNPs (8 wt %)	MS	627	The thermal conductivity increases with GNPs at the loss of Vickers microhardness after 1 wt % of GNP.	[214]
14	Pu et al.	2014	RGO (1 wt %)	Sn + MgSr	21.8	The thermal conductivity decreases after 1 wt % RGO. The silica layer on S-graphene makes electrically conducting graphene insulating, reduces the modulus mismatch between the filler and matrix, and improves the interfacial interactions of the nanocomposites, which results in enhanced thermal conductivity.	[215]
			3-aminopropyl triethoxysilane (APTES) functionalized graphene oxide (A-graphene) (8 wt %)		47.1		
			Silica-coated A-graphene (S-graphene) (8 wt %)		76.5		
15	Fu et al.	2014	Graphite (44.30 wt %)	MS	888.2	The maximum improvement in thermal conductivity was observed in the case of graphene sheets with thickness of 1.5 nm.	[216]
			Graphite nanoflakes (16.81 wt %)		982.3		
			Graphene sheets (10.10 wt %)		2258.8		
16	Li et al.	2014	Aligned MLG (AG) (11.8 wt %)	Sn	16670	The alignment of MLG causes an exceptional improvement in thermal conductivity and exceeds other filler-based epoxy nanocomposites.	[193]
17	Guo and Chen	2014	GNPs (25 wt %)	Sn	780	Ball milling is more effective in improving the thermal conductivity of GNP/epoxy than sonication. The thermal conductivity decreases when ball milling is carried out for more than 30 h.	[126]
			GNPs (25 wt %)	BM	1420		
18	Corcione and Maffezzoli	2013	Natural graphite (NG) (1 wt %)	Sn	24.1	The thermal conductivity decreases with increasing wt % of NG after 1 wt %. The thermal conductivity decreases after 2 wt % of GNPs. The maximum improvement in thermal conductivity was observed with expanded graphite.	[217]
			GNPs (2 wt %)		89.8		
			Expanded graphite (EGS) (3 wt %)		232.1		
19	Chandrasekaran et al.	2013	GNP (2 wt %)	3RM	14	The thermal conductivity increases with increasing temperature.	[73]
20	Min et al.	2013	GNPs (5 wt %)	Sn	240	High aspect ratio of GNPs and oxygen functional groups play a significant role in improving thermal conductivity of nanocomposites.	[218]
21	Hsiao et al.	2013	Silica (1 wt %)	Sn + ShM	19	The existence of the intermediate silica layer enhances the interfacial attractions between TRGO and epoxy and improved dispersion state, which caused a significant increase in thermal conductivity.	[219]
			Thermally reduced graphene oxide (TRGO) (1 wt %)		26.5		
			Silica nanosheets (Silica-NS) (1 wt %)		37.5		
			TRGO-silica-NS (1 wt %)		61.5		
22	Zhou et al.	2013	Untreated GNPs (12 wt %)	Sn + MgSr	139.3	Silane functionalization can significantly improve thermal conductivity of GNP/epoxy.	[220]
			Silane-treated COOH-MWCNTs (6 wt %)		192.9		
			Silane-treated GNPs (6 wt %)		525		
23	Raza et al.	2012	GNPs, 5 µm, 30 wt %, in rubbery epoxy	MS	818.6	The thermal conductivity increases with increasing particle size. The particle size distribution significantly influences the thermal conductivity. GNPs with a broad particle size distribution gave higher thermal conductivity than the particles with a narrow particle size distribution, due to the availability of smaller particles that can bridge gaps between larger particles.	[221]
			GNPs, 5 µm, 20 wt %, in rubbery epoxy	ShM	332.6		
			GNPs, 15 µm, 25 wt %, in rubbery epoxy	MS	1228.4		
			GNPs, 15 µm, 25 wt %, in rubbery epoxy	ShM	1118.2		
			GNPs, 20 µm, 20 wt %, in rubbery epoxy	ShM	684.6		
			GNPs, 20 µm, 12 wt %, in glassy epoxy	ShM	567.6		
			GNPs, 15 µm, 20 wt %, in glassy epoxy	MS	683		
24	Kim et al.	2012	GO (3 wt %)	Sn	90.4	The increase in thermal conductivity decreases with Al(OH)_3_ coating on GO.	[222]
			Al(OH)_3_-coated graphene oxide (Al-GO) (3 wt %)		35.1		
25	Chatterjee et al.	2012	Amine functionalized expanded graphene nanoplatelets (EGNPs) (2 wt %)	Sn + 3RM	36	The EGNPs form a conductive network in the epoxy matrix allowing for increased thermal conductivity.	[83]
26	Im and Kim	2012	Thermally conductive graphene oxide (GO) (50 wt %)	Sn	111	The thermal conductivity decreases after 50 wt %, which can be attributed to residual epoxy that forms an insulting layer on reinforcement. MWCNT helps the formation of 3D network structure.	[223]
			Thermally conductive graphene oxide (GO) (50 wt %), MWCNTs (0.36 wt %)		203.4		
27	Heo et al.	2012	Al_2_O_3_ (80 wt %), GO (5 wt %)	3RM	1,650	The increase in thermal conductivity decreases with Al(OH)_3_ coating of GO.	[224]
			Al(OH)_3_-coated GO (5 wt %)		1,450		
28	Huang et al.	2012	MWNTs (65 wt %)	MS	1,100	GNPs are more effective in improving thermal conductivity than MWNTs. The maximum improvement in thermal conductivity was observed in the case of hybrid fillers.	[225]
			GNPs (65 wt %)		2,750		
			MWNTs (38 wt %), GNPs (38 wt %)		3,600		
29	Teng et al.	2011	MWNT (4 wt %)	Sn	160	GNPs showed a significantly greater increase in thermal conductivity than MWNTs. The maximum improvement in thermal conductivity is shown by non-covalent functionalized GNS, which can be attributed to high surface area and uniform dispersion of GNS.	[114]
			GNPs(4 wt %)		700		
			Poly(glycidyl methacrylate containing localized pyrene groups (Py-PGMA) functionalized GNPs (Py-PGMA-GNS)		860		
30	Gallego et al.	2011	MWNTs (1 wt %) in nanofluids	ShM	66.7	The layered structure of MWNTs enables an efficient phonon transport through the inner layers, while SWNTs present a higher resistance to heat flow at the interface, due to its higher surface area. The f-MWNTs have functional groups on their surface, acting as scattering points for the phonon transport.	[226]
			f-MWNTs (0.6 wt %) in nanofluids		20		
			SWNTs (0.6 wt %) in nanofluids		20		
			Functionalized graphene sheet (FGS) (1 wt %) in nanofluids		0		
			GO (1 wt %) in nanofluids		0		
			MWNTs(1 wt %) in nanocomposites		72.7		
			Functionalized graphene sheet (FGS) (1 wt %) in nanocomposites		63.6		
31	Tien et al.	2011	Graphene flakes (12 wt %)	Sn	350	The thermal conductivity increases exponentially with increasing wt % of graphene flakes.	[227]
32	Ganguli et al.	2008	Exfoliated graphite flakes (20 wt %)	SM	2,087.2	The thermal conductivity increases with chemical functionalization.	[177]
			Chemically functionalized graphite flakes (20 wt %)		2,907.2		
33	Yu et al.	2008	Carbon black (CB) (10 wt %)	Sn + ShM	75	The hybrid filler demonstrates a strong synergistic effect and surpasses the performance of the individual SWNT and GNP filler.	[228]
			SWNTs (10 wt %)		125		
			GNPs (10 wt %)		625		
			GNPs (7.5 wt %), SWNTs (2.5 wt %)		775		

**Table 3:** A brief record of epoxy-based nanocomposites studied for improvement in electrical conductivity values. HSM: high speed mixing.

Sr.	Authors	Year	Reinforcement/wt %	Dispersion method	% Increase in electrical conductivity	Remarks	Ref.
1	Wu et al.	2015	GNPs (1.5 wt %), transverse to alignment	Sn + 3RM	1 × 10^7^	The maximum thermal conductivity was observed in the case of aligned GNPs.	[229]
			GNPs (3 wt %), randomly oriented		1 × 10^8^		
			GNPs (3 wt %), parallel to alignment		1 × 10^10^		
2	Liu et al.	2015	Graphene woven fabric (GWF) (0.62 wt %)	None.	1 × 10^13^	(Samples were produced using resin infiltration.) The average number of graphene layers in GWFs varied between 4 and 12.	[230]
3	Ming et al.	2015	Graphene foam (GF) (80 wt %)	None.	8 × 10^2^	(Samples were produced using hot pressing.) The electrical conductivity of pure graphene foam (GF) is 2.9 S-cm^-1^, which is much lower than graphene, which can be because of the presence of structural defects.	[231]
5	Ghaleb et al.	2014	GNPs (1.1 wt %)	Sn	1.39 × 10^6^	GNPs are more effective in improving the thermal conductivity of epoxy than MWCNTs.	[159]
			MWCNTs (1.9 wt %)		1.62 × 10^5^		
6	Tang et al.	2014	GO (5 wt %)	Sn + HSM	1.92 × 10^9^	The surface functionalization of GO can significantly improve the electrical conductivity of GO–epoxy.	[232]
			Diamine polyetheramine functionalized GO (GO-D230) (5 wt %)		1.92 × 10^12^		
7	Dou et al.	2014	Silver plated graphene (Ag-G) (25 wt %)	Sn + MS	4.13 × 10^2^	Ag–graphene can be used in electronic applications due to its high electrical conductivity.	[233]
8	Tang et al.	2014	GO (3.6 wt %)	Sn	1 × 10^18^	The surface functionalization significantly improves electrical conductivity.	[234]
			Polyetheramine refluxed GO (GO-D2000) (3.6 wt %)		1 × 10^17^		
9	Monti et al.	2013	GNPs (3 wt %)	Sn + MS	2.08 × 10^5^	The samples were produced using chloroform.	[235]
			GNPs (3 wt %)		1.16 × 10^5^	The samples were produced using tetrahydrofuran.			
10	Wajid et al.	2013	GNPs (0.24 wt %)	Sn + MS	2.22 × 10^3^	The samples were produced using dimethylformamide.	[189]
11	Chandrakekaran et al.	2013	GNPs (1 wt %)	Sn + ShM	1 × 10^4^	3RM is more effective in improving the electrical conductivity of epoxy than sonication and high speed shear mixing.	[73]
			GNPs (2 wt %)	3RM	1 × 10^8^		
12	Suherman et al.	2013	GNPs (80 wt %), CNTs (5 wt %), through-plane	BM + MS	7.30 × 10^17^	The electrical conductivity significantly increases with hybrid filler.	[236]
			GNPs (80 wt %), CNTs (5 wt %), in-plane		1.80 × 10^18^		
			GNPs (80 wt %), through-plane		4 × 10^17^		
			GNPs (80 wt %) in-plane		5 × 10^17^		
13	Mancinelli et al.	2013	GO (0.5 wt %)	Sn	240	The conductivity was measured before post-curing.	[237]
			GO (0.5 wt %)		730	The conductivity was measured after post-curing.	
			Octadecylamine (ODA)-treated partially reduced and chemically modified GO (MGO) (0.5 wt %)		550	The conductivity was reduced after functionalization.	
			GO (0.5 wt %)	Two phase extraction	240	The system was not fully cured during curing process.	
			GO (0.5 wt %)		7.80 × 10^3^	The conductivity significantly increased after post-curing.	
14	Al-Ghamdi et al.	2013	Foliated graphite nanosheets (FGNs) (40 wt %)	Centrifugal mixing	9.90 × 10^3^	Dielectric properties of epoxy–FGN composites decreased with an increase in frequency.	[238]
15	Kim et al.	2012	Al(OH)_3_ functionalized GO (Al-GO) (3 wt %)	MS + MgSr	75	The increase in electrical conductivity decreases with Al(OH)_3_ functionalization of GO.	[239]
			GO (3 wt %)		115		
16	Heo et al.	2012	Al_2_O_3_ (80 wt %), Al(OH)_3_ functionalized GO (Al-GO) (5 wt %)	3RM	2.90 × 10^3^	The increase in electrical conductivity with Al(OH)_3_ functionalization decreased. The electrically insulating Al(OH)_3_ on the graphene oxide nanosheet can prevent electron tunneling and act as ion traps which block ion mobility, resulting in a decrease in the electrical properties of nanocomposites.	[224]
			Al_2_O_3_ (80 wt %), GO (5 wt %)		4.90 × 10^3^		
17	Tien et al.	2011	Graphite flakes (14 wt %)	Sn	4 × 10^7^	The percolation threshold was 8 wt %.	[227]
18	Fan et al.	2009	GNPs (5 wt %)	Sn + MS	5.50 × 10^10^	The maximum electrical conductivity was observed in the case of hybrid fillers.	[240]
			GNPs (4.5 wt %), carbon black (CB) (0.5 wt %)		5.50 × 10^12^		
19	Jovic et al.	2008	Expanded graphite (EG) (8 wt %)	Sn	5.50 × 10^17^	The electrical conductivity further increases with the application of electric field.	[241]
20	Li et al.	2007	MWCNTs (1 wt %)	Sn	4.63 × 10^7^	The samples were produced using acetone.	[242]
21	Pecastaings et al.	2004	MWCNTs (20 wt %)	Sn + MS	4.53 × 10^3^	The samples were produced using acetone.	[243]

## Abbreviations

The following abbreviations are used in this manuscript:

3DGN	Three dimensional graphene network
3RM	Three roll milling
A	Aramid fibers
APTS-GO	Amino-functionalized graphene oxide (GO)
ATGO	3-Aminopropyltriethoxysilane functionalized silica nanoparticles attached GO
ATP	Attapulgite
ATS	3-amino functionalized silica nanoparticles
BM	Ball milling
CM	Centrifugal mixing
CNF	Carbon nanofiber
CNFs	Vapor grown carbon nanofibers
CNTs	Carbon nanotubes
DDS	Diaminodiphenylsulfone
DGEBA-f-GO	Diglycidyl ether of bisphenol-A functionalized GO
DRA	Discontinuously reinforced aluminum
DRTi	Discontinously reinforced titanium
EGNPs	Amine functionalized expanded graphene nanoplatelets
EMCs	Epoxy matrix composites
fGnPs	Polybenzimidazole functionalized graphene platelets (GnPs)
GF	Graphene foam
G-NH_2_	Amino-functionalized GNPs
GnPs	Graphene platelets
GNPs*	Graphite nanoplatelets
GNs	Amine functionalized graphene sheets
GNSs	Graphene nanosheets
GO	Graphene oxide
GP	Graphite particles
GPLs	Graphene nanoplatelets
GPTS-GO	Epoxy functionalized GO
G-Si	Silane modified GNPs
HPH + 3RM	High pressure homogenizer + three roll milling
HSM	High speed mixing
m-clay	Surface modified nano clay
m-CNFs	Triazole functionalized carbon nanofibers
MERGO	Microwave exfoliated reduced graphene oxide
m-GnP	Surface modified GnP
m-GnP*	Surfactant modified graphene platelets
m-GP	Surface modified graphene platelets
MgSr	Magnetic stirring
MLG	Multi-layer graphene
MS	Mechanical stirring
MS + USn	Mechanical stirring + Ultrasonication
MWCNTs	Multi-walled carbon nanotubes
MWNTs	Multi-walled carbon nanotubes
ND	Nanodiamond
P	Polyacrylonitrile (PAN) fibers
p-CNFs	Pristine carbon nanofibers
PEA	Polyetheramine
phr	Per hundred parts of resin
PMCs	Polymer matrix composites
Q/I	Quasi-isotropic
RGO	Thermally reduced graphene oxide
SA	Surface area
SATPGO	3-Aminopropyltriethoxysilane modified silica nanoparticles attached graphene oxide
SCFs	Short carbon fibers
ShM	Shear mixing
Silane-f-GO	Silane functionalized GO
SM	Speed mixing
Sn	Sonication
Sn + BM	Sonication + Ball milling
Sn + MgSr	Sonication + Magnetic stirring
Sn + MS	Sonication + Mechanical stirring
SnP	Silver nanoparticles
SnW	Silver nanowires
SWCNTs	Single-walled carbon nanotubes
SWNTs	Single-walled carbon nanotubes
TEM	Transmission electron microscopy
TPE	Two phase extraction
UG	Unmodified graphene nanoplatelets
U-GnP	Unmodified graphene platelets
USn	Ultrasonication

## References

[1] Carlson R.L., Kardomateas G.A., Craig J.I. (2012). Mechanics of Failure Mechanisms in Structures.

[2] Miracle D.B., Donaldson S.L. (2001). ASM Handbook Volume 21: Composites.

[3] Yao X.F., Zhou D., Yeh H.Y. (2008). Macro/microscopic fracture characterizations of SiO_2_/epoxy nanocomposites. Aerosp. Sci. Technol..

[4] Wetzel B., Rosso P., Haupert F., Friedrich K. (2006). Epoxy nanocomposites—Fracture and toughening mechanisms. Eng. Fract. Mech..

[5] Naous W., Yu X.Y., Zhang Q.X., Naito K., Kagawa Y. (2006). Morphology, tensile properties, and fracture toughness of epoxy/Al_2_O_3_ nanocomposites. J. Polym. Sci. Part B.

[6] Kim B.C., Park S.W., Lee D.G. (2008). Fracture toughness of the nano-particle reinforced epoxy composite. Compos. Struct..

[7] Wang K., Chen L., Wu J., Toh M.L., He C., Yee A.F. (2005). Epoxy nanocomposites with highly exfoliated clay: Mechanical properties and fracture mechanisms. Macromolecules.

[8] Liu W., Hoa S.V., Pugh M. (2005). Fracture toughness and water uptake of high-performance epoxy/nanoclay nanocomposites. Compos. Sci. Technol..

[9] Gojny F.H., Wichmann M.H.G., Köpke U., Fiedler B., Schulte K. (2004). Carbon nanotube-reinforced epoxy-composites: Enhanced stiffness and fracture toughness at low nanotube content. Compos. Sci. Technol..

[10] Yu N., Zhang Z.H., He S.Y. (2008). Fracture toughness and fatigue life of MWCNT/epoxy composites. Mater. Sci. Eng. A.

[11] Srikanth I., Kumar S., Kumar A., Ghosal P., Subrahmanyam C. (2012). Effect of amino functionalized MWCNT on the crosslink density, fracture toughness of epoxy and mechanical properties of carbon-epoxy composites. Compos. Part. A Appl. Sci. Manuf..

[12] Mathews M.J., Swanson S.R. (2007). Characterization of the interlaminar fracture toughness of a laminated carbon/epoxy composite. Compos. Sci. Technol..

[13] Arai M., Noro Y., Sugimoto K.I., Endo M. (2008). Mode-I and mode II interlaminar fracture toughness of CFRP laminates toughened by carbon nanofiber interlayer. Compos. Sci. Technol..

[14] Wong D.W.Y., Lin L., McGrail P.T., Peijs T., Hogg P.J. (2010). Improved fracture toughness of carbon fibre/epoxy composite laminates using dissolvable thermoplastic fibres. Compos. Part. A.

[15] Novoselov K.S., Geim A.K., Morozov S.V., Jiang D., Zhang Y., Dubonos S.V., Grigorieva I.V., Firsov A.A. (2004). Electric Field Effect in Atomically Thin Carbon Films. Science.

[16] Pokharel P., Truong Q.-T., Lee D.S. (2014). Multi-step microwave reduction of graphite oxide and its use in the formation of electrically conductive graphene/epoxy composites. Compos. Part B.

[17] Tian M., Qu L., Zhang X., Zhang K., Zhu S., Guo X., Han G., Tang X., Sun Y. (2014). Enhanced mechanical and thermal properties of regenerated cellulose/graphene composite fibers. Carbohydr. Polym..

[18] Xu Z., Zhang J., Shan M., Li Y., Li B., Niu J., Zhou B., Qian X. (2014). Organosilane-functionalized graphene oxide for enhanced antifouling and mechanical properties of polyvinylidene fluoride ultrafiltration membranes. J. Membr. Sci..

[19] Bkakri R., Sayari A., Shalaan E., Wageh S., Al-Ghamdi A.A., Bouazizi A. (2014). Effects of the graphene doping level on the optical and electrical properties of ITO/P3HT:Graphene/Au organic solar cells. Superlattices Microstruct..

[20] Lian Y., He F., Wang H., Tong F. (2014). A new aptamer/graphene interdigitated gold electrode piezoelectric sensor for rapid and specific detection of staphylococcus aureus. Biosens. Bioelectron..

[21] Abdin Z., Alim M.A., Saidur R., Islam M.R., Rashmi W., Mekhilef S., Wadi A. (2013). Solar energy harvesting with the application of nanotechnology. Renew. Sustain. Energy Rev..

[22] Sun W., Hu R., Liu H., Zeng M., Yang L., Wang H., Zhu M. (2014). Embedding nano-silicon in graphene nanosheets by plasma assisted milling for high capacity anode materials in lithium ion batteries. J. Power Sources.

[23] Azeez A.A., Rhee K.Y., Park S.J., Hui D. (2013). Epoxy clay nanocomposites—Processing, properties and applications: A review. Compos. Part. B.

[24] Aziz A., Lim H.N., Girei S.H., Yaacob M.H., Mahdi M.A., Huang N.M., Pandikumar A. (2015). Silver/graphene nanocomposite-modified optical fiber sensor platform for ethanol detection in water medium. Sens. Actuators B Chem..

[25] Agnihotri N., Chowdhury A.D., De A. (2015). Non-enzymatic electrochemical detection of cholesterol using β-cyclodextrin functionalized graphene. Biosens. Bioelectron..

[26] Galpaya D., Wang M., Liu M., Motta N., Waclawik E., Yan C. (2012). Recent Advances in fabrication and characterization of graphene-polymer nanocomposites. Sci. Res..

[27] Shahil K.M.F., Balandin A.A. (2012). Thermal properties of graphene and multilayer graphene: Applications in thermal interface materials. Solid State Commun..

[28] Al-Saleh M.H., Sundararaj U. (2011). Review of the mechanical properties of carbon nanofiber/polymer composites. Compos. Part A.

[29] Sanjinés R., Abad M.D., Vâju C., Smajda R., Mionić M., Magrez A. (2011). Electrical properties and applications of carbon based nanocomposite materials: An overview. Surf. Coat. Technol..

[30] Potts J.R., Dreyer D.R., Bielawski C.W., Ruoff R.S. (2011). Graphene-based polymer nanocomposites. Polymer (Guildf).

[31] Qin F., Brosseau C. (2012). A review and analysis of microwave absorption in polymer composites filled with carbonaceous particles. J. Appl. Phys..

[32] Lee S.-Y., Park S.-J. (2012). Comprehensive review on synthesis and adsorption behaviors of graphene-based materials. Carbon Lett..

[33] Singh V., Joung D., Zhai L., Das S., Khondaker S.I., Seal S. (2011). Graphene-based materials: Past, present and future. Prog. Mater. Sci..

[34] Van Rooyen L.J., Karger-Kocsis J.J., Kock L.D., David Kock L. (2015). Improving the helium gas barrier properties of epoxy coatings through the incorporation of graphene nanoplatelets and the influence of preparation techniques. J. Appl. Polym. Sci..

[35] Kim H., Abdala A.A., Macosko C.W. (2010). Graphene/polymer nanocomposites. Macromolecules.

[36] Dhand V., Rhee K.Y., Kim H.J., Jung D.H. (2015). A comprehensive review of graphene nanocomposites: research status and trends. J. Nanomater..

[37] Santamaria A., Muñoz M.E., Fernández M., Landa M. (2013). Electrically conductive adhesives with a focus on adhesives that contain carbon nanotubes. J. Appl. Polym. Sci..

[38] Yang M.-Q., Xu Y.-J. (2013). Selective photoredox using graphene-based composite photocatalysts. Phys. Chem. Chem. Phys..

[39] Srinivas G., Guo Z.X. (2014). Graphene-based materials: Synthesis and gas sorption, storage and separation. Prog. Mater. Sci..

[40] Xu Z., Chen L., Zhou B., Li Y., Li B., Niu J., Shan M., Guo Q., Wang Z., Qian X. (2013). Nano-structure and property transformations of carbon systems under γ-ray irradiation: A review. RSC Adv..

[41] Hu K., Kulkarni D.D., Choi I., Tsukruk V.V. (2014). Graphene-polymer nanocomposites for structural and functional applications. Prog. Polym. Sci..

[42] Young R.J., Kinloch I.A., Gong L., Novoselov K.S. (2012). The mechanics of graphene nanocomposites: A review. Compos. Sci. Technol..

[43] Zaman I., Manshoor B., Khalid A., Araby S. (2014). From clay to graphene for polymer nanocomposites—A survey. J. Polym. Res..

[44] Sun X., Sun H., Li H., Peng H. (2013). Developing polymer composite materials: Carbon nanotubes or graphene?. Adv. Mater..

[45] Kuilla T., Bhadra S., Yao D., Kim N.H., Bose S., Lee J.H. (2010). Recent advances in graphene-based polymer composites. Prog. Polym. Sci..

[46] Rasheed A., Khalid F.A. (2014). Fabrication and properties of CNTs reinforced polymeric matrix nanocomposites for sports applications. IOP Conf. Ser. Mater. Sci. Eng..

[47] Yue L., Pircheraghi G., Monemian S.A., Manas-Zloczower I. (2014). Epoxy composites with carbon nanotubes and graphene nanoplatelets—Dispersion and synergy effects. Carbon.

[48] Jean-Pierre P., Roberto W. (2010). Epoxy Polymers New Materials and Innovations.

[49] Sanjay M. (2002). Composites Manufacturing Materials, Product, and Process Engineering.

[50] Valery V., Evgeny M. (2001). Mechanics and Analysis of Composite Materials.

[51] Atif R., Inam F. (2016). Influence of macro-topography on damage tolerance and fracture toughness of monolithic epoxy for tribological applications. World J. Eng. Technol..

[52] Wongbong C., Jo-Won L. (2012). Graphene Synthesis and Applications.

[53] Warner J.H., Fransizka S., Mark R., Bachmatiuk A. (2013). Graphene: Fundamentals and Emergent Applications.

[54] Mikhail K., Iosifovich K.M. (2012). Graphene: Carbon in Two Dimensions.

[55] Wolf E.L. (2013). Graphene: A New Paradigm in Condensed Matter and Device Physics.

[56] Quintana M., Spyrou K., Grzelczak M., Browne W.R., Rudolf P., Prato M. (2010). Functionalization of graphene. ACS Nano.

[57] Wang X., Jin J., Song M. (2013). An investigation of the mechanism of graphene toughening epoxy. Carbon.

[58] Jia J., Kan C.-M., Lin X., Shen X., Kim J.-K. (2014). Effects of processing and material parameters on synthesis of monolayer ultralarge graphene oxide sheets. Carbon.

[59] Ma J., Meng Q., Zaman I., Zhu S., Michelmore A., Kawashima N., Wang C. H., Kuan H.-C. (2014). Development of polymer composites using modified, high-structural integrity graphene platelets. Compos. Sci. Technol..

[60] Loomis J., Panchapakesan B. (2012). Dimensional dependence of photomechanical response in carbon nanostructure composites: A case for carbon-based mixed-dimensional systems. Nanotechnology.

[61] Karger-Kocsis J., Mahmood H., Pegoretti A. (2015). Recent advances in fiber/matrix interphase engineering for polymer composites. Prog. Mater. Sci..

[62] Dieter G.E. (1988). Mechanical Metallurgy.

[63] Wan Y.-J., Tang L.-C., Gong L.-X., Yan D., Li Y.-B., Wu L.-B., Jiang J.-X., Lai G.-Q. (2014). Grafting of epoxy chains onto graphene oxide for epoxy composites with improved mechanical and thermal properties. Carbon.

[64] Bindu Sharmila T.K., Nair A.B., Abraham B.T., Beegum P.M.S., Thachil E.T. (2014). Microwave exfoliated reduced graphene oxide epoxy nanocomposites for high performance applications. Polymer (Guildf).

[65] Zhang Y., Wang Y., Yu J., Chen L., Zhu J., Hu Z. (2014). Tuning the interface of graphene platelets/epoxy composites by the covalent grafting of polybenzimidazole. Polymer (Guildf).

[66] Ahmadi-Moghadam B., Sharafimasooleh M., Shadlou S., Taheri F. (2014). Effect of functionalization of graphene nanoplatelets on the mechanical response of graphene/ epoxy composites. Mater. Des..

[67] Chandrasekaran S., Sato N., Tölle F., Mülhaupt R., Fiedler B., Schulte K. (2014). Fracture toughness and failure mechanism of graphene-based epoxy composites. Compos. Sci. Technol..

[68] Wan Y.-J., Gong L.-X., Tang L.-C., Wu L.-B., Jiang J.-X. (2014). Mechanical properties of epoxy composites filled with silane-functionalized graphene oxide. Compos. Part A.

[69] Zaman I., Manshoor B., Khalid A., Meng Q., Araby S. (2014). Interface modification of clay and graphene platelets reinforced epoxy nanocomposites: A comparative study. J. Mater. Sci..

[70] Jiang T., Kuila T., Kim N.H., Lee J.H. (2014). Effects of surface-modified silica nanoparticles attached graphene oxide using isocyanate-terminated flexible polymer chains on the mechanical properties of epoxy composites. J. Mater. Chem. A.

[71] Shokrieh M.M., Ghoreishi S.M., Esmkhani M., Zhao Z. (2014). Effects of graphene nanoplatelets and graphene nanosheets on fracture toughness of epoxy nanocomposites. Fatigue Fract. Eng. Mater. Struct..

[72] Tang L.-C., Wan Y.-J., Yan D., Pei Y.-B., Zhao L., Li Y.-B., Wu L.-B., Jiang J.-X., Lai G.-Q. (2013). The effect of graphene dispersion on the mechanical properties of graphene/epoxy composites. Carbon.

[73] Chandrasekaran S., Seidel C., Schulte K. (2013). Preparation and characterization of graphite nano-platelet (GNP)/epoxy nano-composite: Mechanical, electrical and thermal properties. Eur. Polym. J..

[74] Li Z., Wang R., Young R.J., Deng L., Yang F., Hao L., Jiao W., Liu W. (2013). Control of the functionality of graphene oxide for its application in epoxy nanocomposites. Polymer (Guildf).

[75] Shadlou S., Alishahi E., Ayatollahi M.R. (2013). Fracture behavior of epoxy nanocomposites reinforced with different carbon nano-reinforcements. Compos. Struct..

[76] Jiang T., Kuila T., Kim N.H., Ku B.-C., Lee J.H. (2013). Enhanced mechanical properties of silanized silica nanoparticle attached graphene oxide/epoxy composites. Compos. Sci. Technol..

[77] Liu W., Kong J., Toh W.E., Zhou R., Ding G., Huang S., Dong Y., Lu X. (2013). Toughening of epoxies by covalently anchoring triazole-functionalized stacked-cup carbon nanofibers. Compos. Sci. Technol..

[78] Wang R., Li Z., Liu W., Jiao W., Hao L., Yang F. (2013). Attapulgite–graphene oxide hybrids as thermal and mechanical reinforcements for epoxy composites. Compos. Sci. Technol..

[79] Alishahi E., Shadlou S., Doagou-R S., Ayatollahi M.R. (2013). Effects of carbon nanoreinforcements of different shapes on the mechanical properties of epoxy-based nanocomposites. Macromol. Mater. Eng..

[80] Ma J., Meng Q., Michelmore A., Kawashima N., Izzuddin Z., Bengtsson C., Kuan H.-C. (2013). Covalently bonded interfaces for polymer/graphene composites. J. Mater. Chem. A.

[81] Feng H., Wang X., Wu D. (2013). Fabrication of spirocyclic phosphazene epoxy-based nanocomposites with graphene via exfoliation of graphite platelets and thermal curing for enhancement of mechanical and conductive properties. Ind. Eng. Chem. Res..

[82] Chatterjee S., Nafezarefi F., Tai N.H., Schlagenhauf L., Nüesch F.A., Chu B.T.T. (2012). Size and synergy effects of nanofiller hybrids including graphene nanoplatelets and carbon nanotubes in mechanical properties of epoxy composites. Carbon.

[83] Chatterjee S., Wang J.W., Kuo W.S., Tai N.H., Salzmann C., Li W.L., Hollertz R., Nüesch F.A., Chu B.T.T. (2012). Mechanical reinforcement and thermal conductivity in expanded graphene nanoplatelets reinforced epoxy composites. Chem. Phys. Lett..

[84] Zaman I., Phan T.T., Kuan H.-C., Meng Q., Bao La L.T., Luong L., Youssf O., Ma J. (2011). Epoxy/graphene platelets nanocomposites with two levels of interface strength. Polymer (Guildf).

[85] Rana S., Alagirusamy R., Joshi M. (2011). Development of carbon nanofibre incorporated three phase carbon/epoxy composites with enhanced mechanical, electrical and thermal properties. Compos. Part. A Appl. Sci. Manuf..

[86] Bortz D.R., Merino C., Martin-Gullon I. (2011). Carbon nanofibers enhance the fracture toughness and fatigue performance of a structural epoxy system. Compos. Sci. Technol..

[87] Zhang G., Karger-Kocsis J., Zou J. (2010). Synergetic effect of carbon nanofibers and short carbon fibers on the mechanical and fracture properties of epoxy resin. Carbon.

[88] Fang M., Zhang Z., Li J., Zhang H., Lu H., Yang Y. (2010). Constructing hierarchically structured interphases for strong and tough epoxy nanocomposites by amine-rich graphene surfaces. J. Mater. Chem..

[89] Jana S., Zhong W.-H. (2009). Graphite particles with a “puffed” structure and enhancement in mechanical performance of their epoxy composites. Mater. Sci. Eng. A.

[90] Rafiee M.A., Rafiee J., Srivastava I., Wang Z., Song H., Yu Z.-Z., Koratkar N. (2010). Fracture and fatigue in graphene nanocomposites. Small.

[91] Kuhn H., Medlin D. (2000). ASM Handbook, Volume 8: Mechanical Testing and Evaluation.

[92] Griffith A.A. (1921). The Phenomena of rupture and flow in solids. Philos. Trans. R. Soc. Lond. Ser. A Contain. Pap. Math. Phys. Character.

[93] Zhang W., Srivastava I., Zhu Y.F., Picu C.R., Koratkar N.A. (2009). Heterogeneity in epoxy nanocomposites initiates crazing: Significant improvements in fatigue resistance and toughening. Small.

[94] (1996). ASM Handbook Volume 19: Fatigue and Fracture.

[95] Saharudin M.S., Atif R., Shyha I., Inam F. (2016). The degradation of mechanical properties in polymer nano-composites exposed to liquid media—A review. RSC Adv..

[96] Atif R., Shyha I., Inam F. (2016). The degradation of mechanical properties due to stress concentration caused by retained acetone in epoxy nanocomposites. RSC Adv..

[97] Chen Q., Liu W., Guo S., Zhu S., Li Q., Li X. (2015). Synthesis of well-aligned millimeter-sized tetragon-shaped graphene domains by tuning the copper substrate orientation. Carbon.

[98] Bhushan B. (2010). Springer Handbook of Nanotechnology.

[99] Faber K.T., Evans A.G. (1983). Crack deflection processes—I. Theory. Acta Metall..

[100] Faber K.T., Evans A.G. (1983). Crack deflection processes—II. Experiment. Acta Metall..

[101] Xie F. (2016). A facile strategy for the reduction of graphene oxide and its effect on thermal conductivity of epoxy-based composites. Express Polym. Lett..

[102] Atif R., Inam F. (2016). The dissimilarities between graphene and frame-like structures. Graphene.

[103] Fan B.-B., Guo H.-H., Zhang R., Jia Y., Shi C.-Y. (2014). Structural evolution during the oxidation process of graphite. Chin. Phys. Lett..

[104] Xu Z., Xue K. (2010). Engineering graphene by oxidation: A first-principles study. Nanotechnology.

[105] Kuo W.-S., Tai N.-H., Chang T.-W. (2013). Deformation and fracture in graphene nanosheets. Compos. Part A Appl. Sci. Manuf..

[106] Palmeri M.J., Putz K.W., Brinson L.C. (2010). Sacrificial bonds in stacked-cup carbon nanofibers: Biomimetic toughening mechanisms for composite systems. ACS Nano.

[107] Lee D., Zou X., Zhu X., Seo J.W., Cole J.M., Bondino F., Magnano E., Nair S. K., Su H. (2012). Ultrafast carrier phonon dynamics in NaOH-reacted graphite oxide film. Appl. Phys. Lett..

[108] Shojaee S.A., Zandiatashbar A., Koratkar N., Lucca D.A. (2013). Raman spectroscopic imaging of graphene dispersion in polymer composites. Carbon.

[109] Tamburrano A., Sarasini F., de Bellis G., D’Aloia A.G., Sarto M.S. (2013). The piezoresistive effect in graphene-based polymeric composites. Nanotechnology.

[110] Yang H., Li F., Shan C., Han D., Zhang Q., Niu L., Ivaska A. (2009). Covalent functionalization of chemically converted graphene sheets via silane and its reinforcement. J. Mater. Chem..

[111] Wang G., Shen X., Wang B., Yao J., Park J. (2009). Synthesis and characterisation of hydrophilic and organophilic graphene nanosheets. Carbon.

[112] Samanman S., Numnuam A., Limbut W., Kanatharana P., Thavarungkul P. (2015). Highly-sensitive label-free electrochemical carcinoembryonic antigen immunosensor based on a novel Au nanoparticles–graphene–chitosan nanocomposite cryogel electrode. Anal. Chim. Acta.

[113] Lee S.-Y., Chong M.-H., Park M., Kim H.-Y., Park S.-J. (2014). Effect of chemically reduced graphene oxide on epoxy nanocomposites for flexural behaviors. Carbon Lett..

[114] Teng C.-C., Ma C.-C.M., Lu C.-H., Yang S.-Y., Lee S.-H., Hsiao M.-C., Yen M.-Y., Chiou K.-C., Lee T.-M. (2011). Thermal conductivity and structure of non-covalent functionalized graphene/epoxy composites. Carbon.

[115] Chu K., Li W., Dong H., Tang F. (2012). Modeling the thermal conductivity of graphene nanoplatelets reinforced composites. EPL Europhys. Lett..

[116] Yang S.-Y., Lin W.-N., Huang Y.-L., Tien H.-W., Wang J.-Y., Ma C.-C.M., Li S.-M., Wang Y.-S. (2011). Synergetic effects of graphene platelets and carbon nanotubes on the mechanical and thermal properties of epoxy composites. Carbon.

[117] Pu N.-W., Peng Y.-Y., Wang P.-C., Chen C.-Y., Shi J.-N., Liu Y.-M., Ger M.-D., Chang C.-L. (2014). Application of nitrogen-doped graphene nanosheets in electrically conductive adhesives. Carbon.

[118] Zhao Q., Hao S. (2007). Toughening mechanism of epoxy resins with micro/nano particles. J. Compos. Mater..

[119] Zhao Q., Hoa S., Ouellette P. (2004). Progressive failure of triaxial woven fabric (TWF) composites with open holes. Compos. Struct..

[120] Bastwros M., Kim G.-Y., Zhu C., Zhang K., Wang S., Tang X., Wang X. (2014). Effect of ball milling on graphene reinforced Al6061 composite fabricated by semi-solid sintering. Compos. Part B.

[121] Wu H., Rook B., Drzal L.T. (2013). Dispersion optimization of exfoliated graphene nanoplatelet in polyetherimide nanocomposites: Extrusion, precoating, and solid state ball milling. Polym. Compos..

[122] Yu M., Shao D., Lu F., Sun X., Sun H., Hu T., Wang G., Sawyer S., Qiu H., Lian J. (2013). ZnO/graphene nanocomposite fabricated by high energy ball milling with greatly enhanced lithium storage capability. Electrochem. Commun..

[123] Jiang X., Drzal L.T. (2011). Reduction in percolation threshold of injection molded high-density polyethylene/exfoliated graphene nanoplatelets composites by solid state ball milling and solid state shear pulverization. J. Appl. Polym. Sci..

[124] Wu H., Zhao W., Chen G. (2012). One-pot in situ ball milling preparation of polymer/graphene nanocomposites. J. Appl. Polym. Sci..

[125] Xu J., Jeon I.-Y., Seo J.-M., Dou S., Dai L., Baek J.-B. (2014). Edge-selectively halogenated graphene nanoplatelets (XGnPs, X = Cl, Br, or I) prepared by ball-milling and used as anode materials for lithium-ion batteries. Adv. Mater..

[126] Guo W., Chen G. (2014). Fabrication of graphene/epoxy resin composites with much enhanced thermal conductivity via ball milling technique. J. Appl. Polym. Sci..

[127] Rodriguez A.M., Prieto P., Prato M., Va E. (2014). Exfoliation of graphite with triazine derivatives under ball-milling conditions: Preparation of few-layer graphene via selective noncovalent interactions. ACS Nano.

[128] Xu J., Shui J., Wang J., Wang M., Liu H., Dou S.X., Jeon I. (2014). Sulfur–graphene nanostructured cathodes via ball-milling for high-performance lithium–sulfur batteries. ACS Nano.

[129] Cravotto G., Cintas P. (2010). Sonication-assisted fabrication and post-synthetic modifications of graphene-like materials. Chemistry.

[130] Yi M., Shen Z., Zhang X., Ma S. (2012). Vessel diameter and liquid height dependent sonication-assisted production of few-layer graphene. J. Mater. Sci..

[131] Ciesielski A., Samorì P. (2014). Graphene via sonication assisted liquid-phase exfoliation. Chem. Soc. Rev..

[132] Wang S., Tang L.A.L., Bao Q., Lin M., Deng S., Goh B.M., Loh K. P. (2009). Room-temperature synthesis of soluble carbon nanotubes by the sonication of graphene oxide nanosheets. J. Am. Chem. Soc..

[133] Akhavan O., Ghaderi E., Esfandiar A. (2011). Wrapping bacteria by graphene nanosheets for isolation from environment, reactivation by sonication, and inactivation by near-infrared irradiation. J. Phys. Chem. B.

[134] Polyakova Stolyarova E.Y., Rim K.T., Eom D., Douglass K., Opila R.L., Heinz T.F., Teplyakov A.V., Flynn G.W. (2011). Scanning tunneling microscopy and X-ray photoelectron spectroscopy studies of graphene films prepared by sonication-assisted dispersion. ACS Nano.

[135] Xu P., Loomis J., King B., Panchapakesan B. (2012). Synergy among binary (MWNT, SLG) nano-carbons in polymer nano-composites: A Raman study. Nanotechnology.

[136] Cheng Y.C., Kaloni T.P., Zhu Z.Y., Schwingenschlögl U. (2012). Oxidation of graphene in ozone under ultraviolet light. Appl. Phys. Lett..

[137] Gracia-espino E., Hu G., Shchukarev A., Wa T. (2014). Understanding the interface of six-shell cuboctahedral and icosahedral palladium clusters on reduced graphene oxide: Experimental and theoretical study. J. Am. Chem. Soc..

[138] Velizhanin K.A., Dandu N., Solenov D. (2014). Electromigration of bivalent functional groups on graphene. Phys. Rev. B.

[139] Radovic L.R., Suarez A., Vallejos-Burgos F., Sofo J.O. (2011). Oxygen migration on the graphene surface. 2. Thermochemistry of basal-plane diffusion (hopping). Carbon.

[140] Radovic L.R., Silva-Tapia A.B., Vallejos-Burgos F. (2011). Oxygen migration on the graphene surface. 1. Origin of epoxide groups. Carbon.

[141] Botas C., Álvarez P., Blanco C., Santamaría R., Granda M., Ares P., Rodríguez-Reinoso F., Menéndez R. (2012). The effect of the parent graphite on the structure of graphene oxide. Carbon.

[142] Šljivančanin Ž., Milošević A.S., Popović Z.S., Vukajlović F.R. (2013). Binding of atomic oxygen on graphene from small epoxy clusters to a fully oxidized surface. Carbon.

[143] Ahmed M.S., Han H.S., Jeon S. (2013). One-step chemical reduction of graphene oxide with oligothiophene for improved electrocatalytic oxygen reduction reactions. Carbon.

[144] Yuan F.-Y., Zhang H.-B., Li X., Ma H.-L., Li X.-Z., Yu Z.-Z. (2014). In situ chemical reduction and functionalization of graphene oxide for electrically conductive phenol formaldehyde composites. Carbon.

[145] Jiang X., Nisar J., Pathak B., Zhao J., Ahuja R. (2013). Graphene oxide as a chemically tunable 2-D material for visible-light photocatalyst applications. J. Catal..

[146] Park J.S., Yu L., Lee C.S., Shin K., Han J.H. (2014). Liquid-phase exfoliation of expanded graphites into graphene nanoplatelets using amphiphilic organic molecules. J. Colloid Interface Sci..

[147] Karger-Kocsis J., Friedrich K. (1993). Microstructure-related fracture toughness and fatigue crack growth behaviour in toughened, anhydride-cured epoxy resins. Compos. Sci. Technol..

[148] Karger-Kocsis J., Gremmels J. (2000). Use of hygrothermal decomposed polyester–urethane waste for the impact modification of epoxy resins. J. Appl. Polym. Sci..

[149] Smith G., Bedrov D., Li L., Byutner O. (2002). A molecular dynamics simulation study of the viscoelastic properties of polymer nanocomposites. J. Chem. Phys..

[150] Corcione C.E., Freuli F., Maffezzoli A. (2013). The aspect ratio of epoxy matrix nanocomposites reinforced with graphene stacks. Polym. Eng. Sci..

[151] Ramos-Galicia L., Mendez L.N., Martínez-Hernández A.L., Espindola-Gonzalez A., Galindo-Esquivel I.R., Fuentes-Ramirez R., Velasco-Santos C. (2013). Improved performance of an epoxy matrix as a result of combining graphene oxide and reduced graphene. Int. J. Polym. Sci..

[152] Li Z., Young R.J., Wang R., Yang F., Hao L., Jiao W., Liu W. (2013). The role of functional groups on graphene oxide in epoxy nanocomposites. Polymer (Guildf).

[153] Liu W., Koh K.L., Lu J., Yang L., Phua S., Kong J., Chen Z., Lu X. (2012). Simultaneous catalyzing and reinforcing effects of imidazole-functionalized graphene in anhydride-cured epoxies. J. Mater. Chem..

[154] Yang H., Shan C., Li F., Zhang Q., Han D., Niu L. (2009). Convenient preparation of tunably loaded chemically converted graphene oxide/epoxy resin nanocomposites from graphene oxide sheets through two-phase extraction. J. Mater. Chem..

[155] Galpaya D., Wang M., George G., Motta N., Waclawik E., Yan C. (2014). Preparation of graphene oxide/epoxy nanocomposites with significantly improved mechanical properties. J. Appl. Phys..

[156] Li W., Dichiara A., Bai J. (2013). Carbon nanotube–graphene nanoplatelet hybrids as high-performance multifunctional reinforcements in epoxy composites. Compos. Sci. Technol..

[157] Cao L., Liu X., Na H., Wu Y., Zheng W., Zhu J. (2013). How a bio-based epoxy monomer enhanced the properties of diglycidyl ether of bisphenol A (DGEBA)/graphene composites. J. Mater. Chem. A.

[158] Wan Y.-J., Tang L.-C., Yan D., Zhao L., Li Y.-B., Wu L.-B., Jiang J.-X., Lai G.-Q. (2013). Improved dispersion and interface in the graphene/epoxy composites via a facile surfactant-assisted process. Compos. Sci. Technol..

[159] Ghaleb Z.A., Mariatti M., Ariff Z.M. (2014). Properties of graphene nanopowder and multi-walled carbon nanotube-filled epoxy thin-film nanocomposites for electronic applications: The effect of sonication time and filler loading. Compos. Part A.

[160] King J.A., Klimek D.R., Miskioglu I., Odegard G.M. (2013). Mechanical properties of graphene nanoplatelet/epoxy composites. J. Appl. Polym. Sci..

[161] Wang X., Song L., Pornwannchai W., Hu Y., Kandola B. (2013). The effect of graphene presence in flame retarded epoxy resin matrix on the mechanical and flammability properties of glass fiber-reinforced composites. Compos. Part A.

[162] Serena Saw W.P., Mariatti M. (2011). Properties of synthetic diamond and graphene nanoplatelet-filled epoxy thin film composites for electronic applications. J. Mater. Sci. Mater. Electron..

[163] Zaman I., Kuan H.-C., Meng Q., Michelmore A., Kawashima N., Pitt T., Zhang L., Gouda S., Luong L., Ma J. (2012). A Facile Approach to Chemically Modified Graphene and its Polymer Nanocomposites. Adv. Funct. Mater..

[164] Hsu C.-H., Hsu M.-H., Chang K.-C., Lai M.-C., Liu P.-J., Chuang T.-L., Yeh J.-M., Liu W.-R. (2014). Physical study of room-temperature-cured epoxy/thermally reduced graphene oxides with various contents of oxygen-containing groups. Polym. Int..

[165] Yang Y., Rigdon W., Huang X., Li X. (2013). Enhancing graphene reinforcing potential in composites by hydrogen passivation induced dispersion. Sci. Rep..

[166] Naebe M., Wang J., Amini A., Khayyam H., Hameed N., Li L.H., Chen Y., Fox B. (2014). Mechanical property and structure of covalent functionalised graphene/epoxy nanocomposites. Sci. Rep..

[167] Qi B., Yuan Z., Lu S., Liu K., Li S., Yang L., Yu J. (2014). Mechanical and thermal properties of epoxy composites containing graphene oxide and liquid crystalline epoxy. Fibers Polym..

[168] Ren F., Zhu G., Ren P., Wang Y., Cui X. (2014). In situ polymerization of graphene oxide and cyanate ester–epoxy with enhanced mechanical and thermal properties. Appl. Surf. Sci..

[169] Qi B. (2014). Enhanced thermal and mechanical properties of epoxy composites by mixing thermotropic liquid crystalline epoxy grafted graphene oxide. Express Polym. Lett..

[170] Lu S., Li S., Yu J., Yuan Z., Qi B. (2013). Epoxy nanocomposites filled with thermotropic liquid crystalline epoxy grafted graphene oxide. RSC Adv..

[171] Shen X.-J., Liu Y., Xiao H.-M., Feng Q.-P., Yu Z.-Z., Fu S.-Y. (2012). The reinforcing effect of graphene nanosheets on the cryogenic mechanical properties of epoxy resins. Compos. Sci. Technol..

[172] Bao C., Guo Y., Song L., Kan Y., Qian X., Hu Y. (2011). In situ preparation of functionalized graphene oxide/epoxy nanocomposites with effective reinforcements. J. Mater. Chem..

[173] Meng Q., Jin J., Wang R., Kuan H.-C., Ma J., Kawashima N., Michelmore A., Zhu S., Wang C.H. (2014). Processable 3-nm thick graphene platelets of high electrical conductivity and their epoxy composites. Nanotechnology.

[174] Atif R., Shyha I., Inam F. (2016). Modeling and experimentation of multi-layered nanostructured graphene-epoxy nanocomposites for enhanced thermal and mechanical properties. J. Compos. Mater..

[175] Yu A., Ramesh P., Itkis M.E., Bekyarova E., Haddon R.C. (2007). Graphite nanoplatelet—epoxy composite thermal interface materials. J. Phys. Chem. C.

[176] Yavari F., Fard H.R., Pashayi K., Rafiee M.a., Zamiri A., Yu Z., Ozisik R., Borca-Tasciuc T., Koratkar N. (2011). Enhanced thermal conductivity in a nanostructured phase change composite due to low concentration graphene additives. J. Phys. Chem. C.

[177] Ganguli S., Roy A.K., Anderson D.P. (2008). Improved thermal conductivity for chemically functionalized exfoliated graphite/epoxy composites. Carbon.

[178] Fukushima H., Drzal L.T., Rook B.P., Rich M.J. (2006). Thermal conductivity of exfoliated graphite nanocomposites. J. Therm. Anal. Calorim..

[179] Xie S.H., Liu Y.Y., Li J.Y. (2008). Comparison of the effective conductivity between composites reinforced by graphene nanosheets and carbon nanotubes. Appl. Phys. Lett..

[180] Lin W., Zhang R., Wong C.P. (2010). Modeling of thermal conductivity of graphite nanosheet composites. J. Electron. Mater..

[181] Nan C.-W., Birringer R., Clarke D.R., Gleiter H. (1997). Effective thermal conductivity of particulate composites with interfacial thermal resistance. J. Appl. Phys..

[182] Shiu S.-C., Tsai J.-L. (2014). Characterizing thermal and mechanical properties of graphene/epoxy nanocomposites. Compos. Part B.

[183] Yu G., Wu P. (2014). Effect of chemically modified graphene oxide on the phase separation behaviour and properties of an epoxy/polyetherimide binary system. Polym. Chem..

[184] Liu T., Zhao Z., Tjiu W.W., Lv J., Wei C. (2014). Preparation and characterization of epoxy nanocomposites containing surface-modified graphene oxide. J. Appl. Polym. Sci..

[185] Liu F., Guo K. (2014). Reinforcing epoxy resin through covalent integration of functionalized graphene nanosheets. Polym. Adv. Technol..

[186] Guan L.-Z., Wan Y.-J., Gong L.-X., Yan D., Tang L.-C., Wu L.-B., Jiang J.-X., Lai G.-Q. (2014). Toward effective and tunable interphases in graphene oxide/epoxy composites by grafting different chain lengths of polyetheramine onto graphene oxide. J. Mater. Chem. A.

[187] Martin-Gallego M., Bernal M.M., Hernandez M., Verdejo R., Lopez-Manchado M.A. (2013). Comparison of filler percolation and mechanical properties in graphene and carbon nanotubes filled epoxy nanocomposites. Eur. Polym. J..

[188] Ribeiro H., Silva W.M., Rodrigues M.-T.F., Neves J.C., Paniago R., Fantini C., Calado H.D.R., Seara L.M., Silva G.G. (2013). Glass transition improvement in epoxy/graphene composites. J. Mater. Sci..

[189] Wajid A.S., Ahmed H.S.T., Das S., Irin F., Jankowski A.F., Green M.J. (2013). High-Performance Pristine Graphene/Epoxy Composites With Enhanced Mechanical and Electrical Properties. Macromol. Mater. Eng..

[190] Zhang X., Alloul O., He Q., Zhu J., Verde M.J., Li Y., Wei S., Guo Z. (2013). Strengthened magnetic epoxy nanocomposites with protruding nanoparticles on the graphene nanosheets. Polymer (Guildf).

[191] Wang X., Xing W., Feng X., Yu B., Song L., Hu Y. (2014). Functionalization of graphene with grafted polyphosphamide for flame retardant epoxy composites: Synthesis, flammability and mechanism. Polym. Chem..

[192] Hu L., Desai T., Keblinski P. (2011). Thermal transport in graphene-based nanocomposite. J. Appl. Phys..

[193] Li Q., Guo Y., Li W., Qiu S., Zhu C., Wei X., Chen M., Liu C., Liao S., Gong Y., Mishra A. K., Liu L. (2014). Ultrahigh Thermal Conductivity of Assembled Aligned Multilayer Graphene/Epoxy Composite. Chem. Mater..

[194] Geim A.K. (2009). Graphene: Status and Prospects. Science.

[195] Geim A.K., Novoselov K.S. (2007). The rise of graphene. Nat. Mater..

[196] Yan W., He W.-Y., Chu Z.-D., Liu M., Meng L., Dou R.-F., Zhang Y., Liu Z., Nie J.-C., He L. (2013). Strain and curvature induced evolution of electronic band structures in twisted graphene bilayer. Nat. Commun..

[197] Castro Neto A.H., Peres N.M.R., Novoselov K.S., Geim A.K., Guinea F. (2009). The electronic properties of graphene. Rev. Mod. Phys..

[198] Zhang Y., Tan Y.-W., Stormer H. L., Kim P. (2005). Experimental observation of the quantum Hall effect and Berry’s phase in graphene. Nature.

[199] Novoselov K.S., Geim A.K., Morozov S.V., Jiang D., Katsnelson M.I., Grigorieva I.V., Dubonos S.V., Firsov A.A. (2005). Two-dimensional gas of massless Dirac fermions in graphene. Nature.

[200] Zhao L., Levendorf M., Goncher S., Schiros T., Pálová L., Zabet-Khosousi A., Rim K. T., Gutiérrez C., Nordlund D., Jaye C., Hybertsen M., Reichman D., Flynn G. W., Park J., Pasupathy A. N. (2013). Local atomic and electronic structure of boron chemical doping in monolayer graphene. Nano Lett..

[201] Han W., Kawakami R.K., Gmitra M., Fabian J. (2014). Graphene spintronics. Nat. Nanotechnol..

[202] Kandare E., Khatibi A.A., Yoo S., Wang R., Ma J., Olivier P., Gleizes N., Wang C.H. (2015). Improving the through-thickness thermal and electrical conductivity of carbon fibre/epoxy laminates by exploiting synergy between graphene and silver nano-inclusions. Compos. Part A.

[203] Tang B., Hu G., Gao H., Hai L. (2015). Application of graphene as filler to improve thermal transport property of epoxy resin for thermal interface materials. Int. J. Heat Mass Transf..

[204] Burger N., Laachachi A., Mortazavi B., Ferriol M., Lutz M., Toniazzo V., Ruch D. (2015). Alignments and network of graphite fillers to improve thermal conductivity of epoxy-based composites. Int. J. Heat Mass Transf..

[205] Zeng C., Lu S., Xiao X., Gao J., Pan L., He Z., Yu J. (2014). Enhanced thermal and mechanical properties of epoxy composites by mixing noncovalently functionalized graphene sheets. Polym. Bull..

[206] Wang F., Drzal L.T., Qin Y., Huang Z. (2014). Mechanical properties and thermal conductivity of graphene nanoplatelet/epoxy composites. J. Mater. Sci..

[207] Zhou T., Nagao S., Sugahara T., Koga H., Nogi M., Suganuma K., Nge T.T., Nishina Y. (2015). Facile identification of the critical content of multi-layer graphene oxide for epoxy composite with optimal thermal properties. RSC Adv..

[208] Zeng C., Lu S., Song L., Xiao X., Gao J., Pan L., He Z., Yu J. (2015). Enhanced thermal properties in a hybrid graphene–alumina filler for epoxy composites. RSC Adv..

[209] Tang D., Su J., Yang Q., Kong M., Zhao Z., Huang Y., Liao X., Liu Y. (2015). Preparation of alumina-coated graphite for thermally conductive and electrically insulating epoxy composites. RSC Adv..

[210] Pan L., Ban J., Lu S., Chen G., Yang J., Luo Q., Wu L., Yu J. (2015). Improving thermal and mechanical properties of epoxy composites by using functionalized graphene. RSC Adv..

[211] Wang R., Zhuo D., Weng Z., Wu L., Cheng X., Zhou Y., Wang J., Xuan B. (2015). A novel nanosilica/graphene oxide hybrid and its flame retarding epoxy resin with simultaneously improved mechanical, thermal conductivity, and dielectric properties. J. Mater. Chem. A.

[212] Zha J.-W., Zhu T.-X., Wu Y.-H., Wang S.-J., Li R.K.Y., Dang Z.-M. (2015). Tuning of thermal and dielectric properties for epoxy composites filled with electrospun alumina fibers and graphene nanoplatelets through hybridization. J. Mater. Chem. C.

[213] Zhou T. (2015). Targeted kinetic strategy for improving the thermal conductivity of epoxy composite containing percolating multi-layer graphene oxide chains. Express Polym. Lett..

[214] Wang Y., Yu J., Dai W., Song Y., Wang D., Zeng L., Jiang N. (2015). Enhanced Thermal and Electrical Properties of Epoxy Composites Reinforced With Graphene Nanoplatelets. Polym. Compos..

[215] Pu X., Zhang H.-B., Li X., Gui C., Yu Z.-Z. (2014). Thermally conductive and electrically insulating epoxy nanocomposites with silica-coated graphene. RSC Adv..

[216] Fu Y.-X., He Z.-X., Mo D.-C., Lu S.-S. (2014). Thermal conductivity enhancement of epoxy adhesive using graphene sheets as additives. Int. J. Therm. Sci..

[217] Esposito Corcione C., Maffezzoli A. (2013). Transport properties of graphite/epoxy composites: Thermal, permeability and dielectric characterization. Polym. Test..

[218] Min C., Yu D., Cao J., Wang G., Feng L. (2013). A graphite nanoplatelet/epoxy composite with high dielectric constant and high thermal conductivity. Carbon.

[219] Hsiao M.-C., Ma C.-C.M., Chiang J.-C., Ho K.-K., Chou T.-Y., Xie X. (2013). Thermally conductive and electrically insulating epoxy nanocomposites with thermally reduced graphene oxide-silica hybrid nanosheets. Nanoscale.

[220] Zhou T., Wang X., Cheng P., Wang T., Xiong D., Wang X. (2013). Improving the thermal conductivity of epoxy resin by the addition of a mixture of graphite nanoplatelets and silicon carbide microparticles. Express Polym. Lett..

[221] Raza M.A., Westwood A.V.K., Stirling C. (2012). Effect of processing technique on the transport and mechanical properties of graphite nanoplatelet/rubbery epoxy composites for thermal interface applications. Mater. Chem. Phys..

[222] Kim J., Yim B., Kim J., Kim J. (2012). The effects of functionalized graphene nanosheets on the thermal and mechanical properties of epoxy composites for anisotropic conductive adhesives (ACAs). Microelectron. Reliab..

[223] Im H., Kim J. (2012). Thermal conductivity of a graphene oxide–carbon nanotube hybrid/epoxy composite. Carbon.

[224] Heo Y., Im H., Kim J., Kim J. (2012). The influence of Al(OH)_3_-coated graphene oxide on improved thermal conductivity and maintained electrical resistivity of Al_2_O_3_/epoxy composites. J. Nanopart. Res..

[225] Huang X., Zhi C., Jiang P. (2012). Toward Effective Synergetic Effects from Graphene Nanoplatelets and Carbon Nanotubes on Thermal Conductivity of Ultrahigh Volume Fraction Nanocarbon Epoxy Composites. J. Phys. Chem. C.

[226] Martin-gallego M., Verdejo R., Khayet M., Maria J., De Zarate O., Essalhi M., Lopez-manchado M.A. (2011). Thermal conductivity of carbon nanotubes and graphene in epoxy nanofluids and nanocomposites. Nanoscale Res. Lett..

[227] Tien D.H., Joonkyu P., Sang A.H., Muneer A., Yongho S., Koo S. (2011). Electrical and Thermal Conductivities of Stycast 1266 Epoxy/Graphite Composites. J. Korean Phys. Soc..

[228] Yu A., Ramesh P., Sun X., Bekyarova E., Itkis M.E., Haddon R.C. (2008). Enhanced thermal conductivity in a hybrid graphite nanoplatelet—Carbon nanotube filler for epoxy composites. Adv. Mater..

[229] Wu S., Ladani R.B., Zhang J., Bafekrpour E., Ghorbani K., Mouritz A.P., Kinloch A.J., Wang C.H. (2015). Aligning multilayer graphene flakes with an external electric field to improve multifunctional properties of epoxy nanocomposites. Carbon.

[230] Liu X., Sun X., Wang Z., Shen X., Wu Y., Kim J.-K. (2015). Planar Porous Graphene Woven Fabric/Epoxy Composites with Exceptional Electrical, Mechanical Properties, and Fracture Toughness. ACS Appl. Mater. Interfaces.

[231] Ming P., Zhang Y., Bao J., Liu G., Li Z., Jiang L., Cheng Q. (2015). Bioinspired highly electrically conductive graphene–epoxy layered composites. RSC Adv..

[232] Tang G., Jiang Z.-G., Li X., Zhang H.-B., Hong S., Yu Z.-Z. (2014). Electrically conductive rubbery epoxy/diamine-functionalized graphene nanocomposites with improved mechanical properties. Compos. Part B Eng..

[233] Dou S., Qi J., Guo X., Yu C. (2014). Preparation and adhesive performance of electrical conductive epoxy-acrylate resin containing silver-plated graphene. J. Adhes Sci. Technol..

[234] Tang G., Jiang Z.-G., Li X., Zhang H.-B., Yu Z.-Z. (2014). Simultaneous functionalization and reduction of graphene oxide with polyetheramine and its electrically conductive epoxy nanocomposites. Chin. J. Polym. Sci..

[235] Monti M., Rallini M., Puglia D., Peponi L., Torre L., Kenny J.M. (2013). Morphology and electrical properties of graphene–epoxy nanocomposites obtained by different solvent assisted processing methods. Compos. Part A Appl. Sci. Manuf..

[236] Suherman H., Sulong A.B., Sahari J. (2013). Effect of the compression molding parameters on the in-plane and through-plane conductivity of carbon nanotubes/graphite/epoxy nanocomposites as bipolar plate material for a polymer electrolyte membrane fuel cell. Ceram. Int..

[237] Mancinelli P., Heid T.F., Fabiani D., Saccani A., Toselli M., Frechette M.F., Savoie S., David E. Electrical conductivity of graphene-based epoxy nanodielectrics. Proceedings of the 2013 Annual Report Conference on Electrical Insulation and Dielectric Phenomena.

[238] Al-Ghamdi A.A., Al-Hartomy O.A., Al-Solamy F., Al-Ghamdi A.A., El-Tantawy F. (2013). Electromagnetic wave shielding and microwave absorbing properties of hybrid epoxy resin/foliated graphite nanocomposites. J. Appl. Polym. Sci..

[239] Kim J., Im H., Kim J., Kim J. (2011). Thermal and electrical conductivity of Al(OH)_3_ covered graphene oxide nanosheet/epoxy composites. J. Mater. Sci..

[240] Fan Z., Zheng C., Wei T., Zhang Y., Lu G. (2009). Effect of Carbon Black on Electrical Property of Graphite Nanoplatelets/Epoxy Resin Composites. Polym. Eng. Sci..

[241] Jović N., Dudić D., Montone A., Antisari M.V., Mitrić M., Djoković V. (2008). Temperature dependence of the electrical conductivity of epoxy/expanded graphite nanosheet composites. Scr. Mater..

[242] Li J., Ma P.C., Chow W.S., To C.K., Tang B.Z., Kim J.-K. (2007). Correlations between Percolation Threshold, Dispersion State, and Aspect Ratio of Carbon Nanotubes. Adv. Funct. Mater..

[243] Sandler J., Shaffer M.S., Prasse T., Bauhofer W., Schulte K., Windle A. (1999). Development of a dispersion process for carbon nanotubes in an epoxy matrix and the resulting electrical properties. Polymer (Guildf).

[244] Raza M.A., Westwood A., Stirling C. (2012). Effect of processing technique on the transport and mechanical properties of vapour grown carbon nanofibre/rubbery epoxy composites for electronic packaging applications. Carbon.

[245] Mas B., Fernández-Blázquez J.P., Duval J., Bunyan H., Vilatela J.J. (2013). Thermoset curing through Joule heating of nanocarbons for composite manufacture, repair and soldering. Carbon.

[246] Chang K.-C., Hsu M.-H., Lu H.-I., Lai M.-C., Liu P.-J., Hsu C.-H., Ji W.-F., Chuang T.-L., Wei Y., Yeh J.-M., Liu W.-R. (2014). Room-temperature cured hydrophobic epoxy/graphene composites as corrosion inhibitor for cold-rolled steel. Carbon.

[247] Prolongo S.G., Jiménez-Suárez A., Moriche R., Ureña A. (2014). Graphene nanoplatelets thickness and lateral size influence on the morphology and behavior of epoxy composites. Eur. Polym. J..

[248] Prolongo S.G., Moriche R., Jiménez-Suárez A., Sanchez M., Ureña A. (2014). Advantages and disadvantages of the addition of graphene nanoplatelets to epoxy resins. Eur. Polym. J..

